# Insect herbivory on *Catula gettyi* gen. et sp. nov. (Lauraceae) from the Kaiparowits Formation (Late Cretaceous, Utah, USA)

**DOI:** 10.1371/journal.pone.0261397

**Published:** 2022-01-21

**Authors:** S. Augusta Maccracken, Ian M. Miller, Kirk R. Johnson, Joseph M. Sertich, Conrad C. Labandeira

**Affiliations:** 1 Department of Earth Sciences, Denver Museum of Nature & Science, Denver, CO, United States of America; 2 Department of Paleobiology, National Museum of Natural History, Smithsonian Institution, Washington, DC, United States of America; 3 Department of Entomology, University of Maryland, College Park, MD, United States of America; 4 National Geographic Society, Washington, DC, United States of America; 5 BEES Program, University of Maryland, College Park, MD, United States of America; 6 College of Life Sciences, Capital Normal University, Beijing,China; Baylor University, UNITED STATES

## Abstract

The Upper Cretaceous (Campanian Stage) Kaiparowits Formation of southern Utah, USA, preserves abundant plant, invertebrate, and vertebrate fossil taxa. Taken together, these fossils indicate that the ecosystems preserved in the Kaiparowits Formation were characterized by high biodiversity. Hundreds of vertebrate and invertebrate species and over 80 plant morphotypes are recognized from the formation, but insects and their associations with plants are largely undocumented. Here, we describe a new fossil leaf taxon, *Catula gettyi* gen et. sp. nov. in the family Lauraceae from the Kaiparowits Formation. *Catula gettyi* occurs at numerous localities in this deposit that represent ponded and distal floodplain environments. The type locality for *C*. *gettyi* has yielded 1,564 fossil leaf specimens of this species, which provides the opportunity to circumscribe this new plant species. By erecting this new genus and species, we are able to describe ecological associations on *C*. *gettyi* and place these interactions within a taxonomic context. We describe an extensive archive of feeding damage on *C*. *gettyi* caused by herbivorous insects, including more than 800 occurrences of insect damage belonging to five functional feeding groups indicating that insect-mediated damage on this taxon is both rich and abundant. *Catula gettyi* is one of the best-sampled host plant taxa from the Mesozoic Era, a poorly sampled time interval, and its insect damage is comparable to other Lauraceae taxa from the younger Late Cretaceous Hell Creek Flora of North Dakota, USA.

## Introduction

Lauraceae Juss. (Order Laurales) is a speciose and anatomically diverse family of aromatic magnoliid angiosperms. Today, the family is generally thought to consist of 45 genera and 2,850 species [1] to perhaps as many as 52 genera and 3,500 species [2]. The Lauraceae are almost exclusively trees and shrubs, although species in the genus *Cassytha* L. may exhibit herbaceous or parasitic growth forms [2,3]. The family is mostly evergreen and presently occupies tropical and warm-temperate forests across a significant altitudinal range [4,5]. Leaves of Lauraceae are often dark green and glossy on their adaxial surfaces and villous and grey-green on their abaxial surfaces [2]. Notably, the leaves are often leathery, which improves their preservation potential in the fossil record [6].

The Lauraceae lineage can be traced to the Early Cretaceous. The oldest unequivocal occurrence of reproductive organs attributed to the Lauraceae are charcoalified flowers of *Potomacanthus lobatus* von Balthazar et al. [7] from the Potomac Group, a Lower Cretaceous deposit (Albian, ca. 108 Ma), from eastern North America. The oldest fossil leaves assigned to Lauraceae are of similar age. They include examples such as those from the Dakota Formation (Albian, ca. 102 Ma), described as *Rogersia dakotensis* Wang and Dilcher [8], *Wolfiophyllum heigii* Wang and Dilcher [8], *Pandemophyllum* Upchurch and Dilcher [9], and *Pabiana* Upchurch and Dilcher [9]. In Upper Cretaceous strata, fossil occurrences of Lauraceae are worldwide. Notable examples include 1) charcoalified flowers, peduncles, fruits, and stems of *Mauldinia* sp. from the Vocontian Basin in southeastern France (Cenomanian, ca. 97 Ma) [10]; 2) carbonized flowers and inflorescences of *Mauldinia bohemica* Eklund and Kvaček [11] from the Peruc–Korycany Formation (Cenomanian, ca. 95 Ma) in the Czech Republic; 3) carbonized flowers of *Perseanthus crossmanensis* Herendeen et al. [12] of the Raritan Formation (Turonian, ca. 91 Ma), New Jersey, U.S.A.; 4) wood of *Paraphyllanthoxylon vancouverense* Jud et al. [13] from the Comox Formation (Coniacian, ca. 89 Ma) in British Columbia, Canada; and 5) leaves of *Cinnamomoides newberryi* Berry [14] from the Hidden Lake Formation of Antarctica (Coniacian, ca. 88.7–86.4 Ma) [15]. The trend of global distribution of the Lauraceae continues into the Cretaceous. For example, the wood of *Sassafrasoxylon gottwaldii* Poole et al. [16] from the Santonian–Maastrichtian López de Bertodano and Santa Marta Formations of Antarctica, lauraceous flowers from the Maastrichtian (ca. 70 Ma) Taratu Formation of New Zealand, and *Marmarthia trivialis* and *M*. *pearsonii* Johnson from the Hell Creek Formation (ca. 67.5–66 Ma) of the Williston Basin, North Dakota, USA [17].

Alongside inferences from molecular diversification proxies [18–20], fossil occurrences of Lauraceae indicate that the family evolved and began to diversify during the Early Cretaceous [ex. 21,22]. Furthermore, fossil attributions indicate that the family was substantially diverse by the end of the Cretaceous [21]. However, evidence for Cretaceous diversification of the family is limited compared to the Cenozoic diversity of Lauraceae [12]. Additional research on the taxonomy and ecological associations of Cretaceous Lauraceae would assist paleobotanists to map the evolution of this important angiosperm family in time and space.

Through several multi-year field campaigns since the 1990s, the biota of the Kaiparowits Formation (Upper Cretaceous, 76.6–74.5 Ma) has been increasingly well known [23–27]. Fossils from this formation number in the thousands and are present in several major museum collections in the United States (e.g. Denver Museum of Nature & Science, Natural History Museum of Utah). Dinosaurian and associated vertebrate fauna, as well as aquatic and infaunal invertebrates, have been extensively described in *At the Top of the Grand Staircase*: *The Late Cretaceous of Southern Utah*, edited by Titus and Loewen [25]. Large collections of megafossil plants, including leaves and wood, and palynoflora have been collected and are presently being described [26]. The stratigraphy, sedimentology, and geochronology of the formation are increasingly well understood [28–30]. Despite this growing body of work, insects, the most diverse group of macroorganisms and a cornerstone of terrestrial ecosystems, have received minimal attention in this formation [31,32]. Indeed, insect body fossils are poorly known worldwide from the Campanian (83.6–72.1 Ma) [33], particularly when compared with insect amber and compression–impression deposits from ca. 120 to 90 Ma [34,35] and the Campanian trace fossil record of insects is nascent in scope [36–39]. With the exception of a newly described moth leaf mine, social insect nests, and dermestid beetle bone borings [31,32,40], the diversity and ecological roles of Kaiparowits Formation insects, such as detritivores, predators and their prey, parasitoids, and herbivores are largely unknown. The trace fossil record of insects in the Kaiparowits Formation can provide independent evidence of insects when the body fossil record is sparse and, moreover, provide novel information on the ecologies of ancient plants and their associations with insects [41].

Fossil plant material, most commonly insect damaged leaves, is the basis for reconstructing the arthropod diversity of ancient terrestrial landscapes and their ecological interactions. The fossil record of plant–arthropod associations is comprised of mimicry [ex. 42], mutualisms [43,44], notably insect pollination [ex. 45–48], and insect herbivory, which is among the most dynamic and copious of these associations [see 49]. Evidence for the damage that herbivorous insects inflict—the punctures, skeletonization, galls and leaf mines in fossil leaves—constitute one of the richest ecological sources of evidence available on species interactions of any kind from the distant past [50]. In particular, understanding the suite of damage that exists on a particular plant species can provide insights into its physical and chemical defenses, ancient food-web structure [ex. 50–52], and the coevolutionary arms race between a plant species and their insect herbivores [53].

The first paleobotanical exploration of the Kaiparowits Formation began in the late 1990s [26]. The approach taken by the Denver Museum of Nature & Science (DMNS) team was to explore and extensively quarry sites with well-preserved fossil leaves to build a comprehensive collection of plant taxa as a baseline for future work. One highly productive locality (Lost Valley, DMNH loc. 4150), yielded more than 4,000 identifiable leaf fossils, all of which were collected by and housed at DMNS. This collection included more than 1,500 specimens of the new taxon described in this paper, providing a rare opportunity to analyze insect damage on a very large sample of leaves from a single species. Using these fossils, we describe a new species within the family Lauraceae, which is the first taxonomic description of a fossil plant taxon from the Kaiparowits and first evidence for Lauraceae occurring in the formation, as well as document the evidence for plant–insect interactions on this new species as an indicator for the diversity and intensity of insect herbivory within the middle Kaiparowits ecosystem. The aims for this study are threefold: 1) Describe and name the new taxon based on fossil leaves; 2) measure the diversity and intensity of insect damage on the new taxon; and 3) compare the diversity and intensity of insect damage to that of other Late Cretaceous taxa attributed to Lauraceae.

## Geologic setting and age

**Fig 1:**
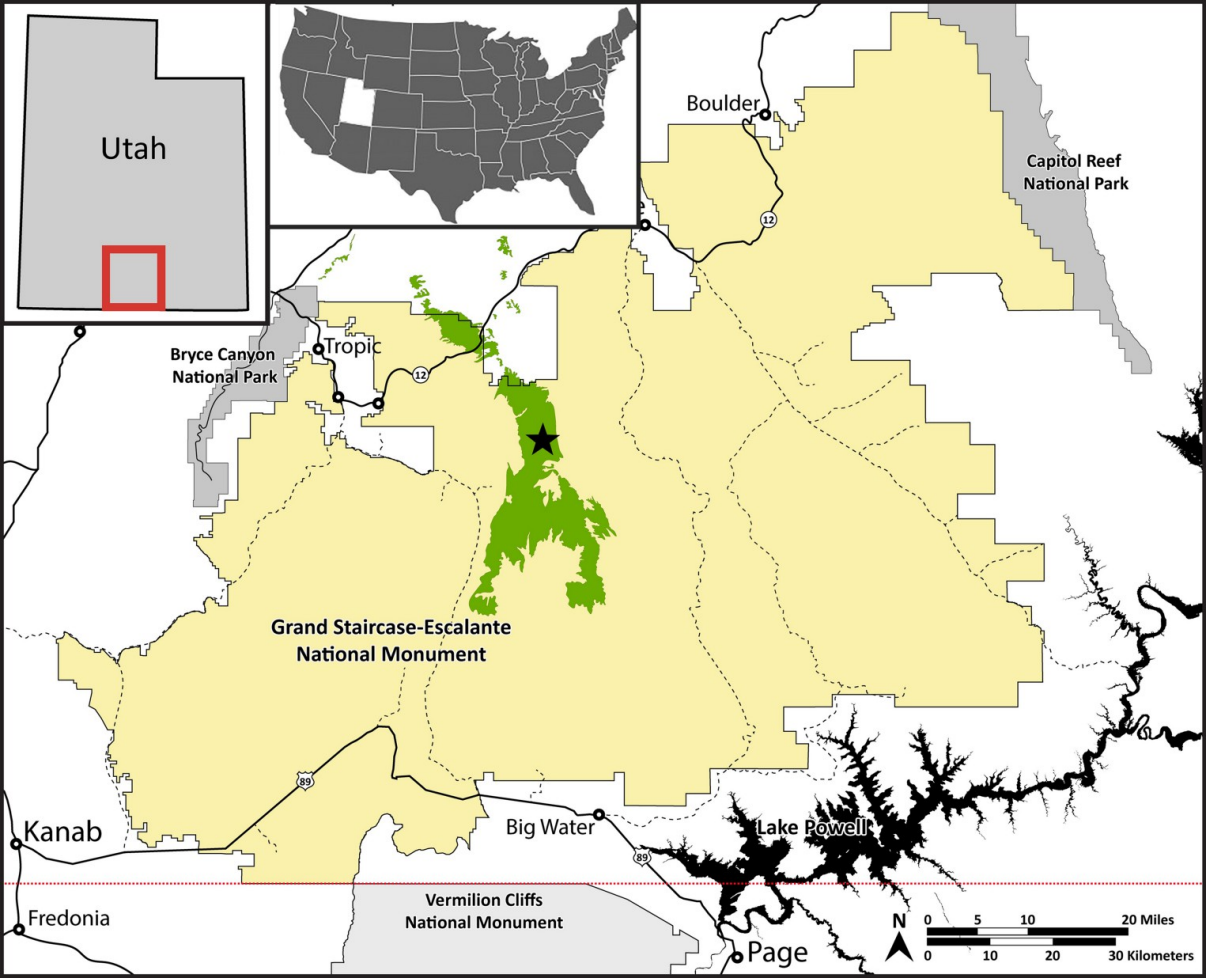
Map of the Grand Staircase-Escalante National Monument. The Kaiparowits Formation outcrop is green. Solid yellow denotes newly reestablished monument boundaries. DMNH loc. 4150, the Lost Valley locality, is denoted by a star. Adapted from Crystal et al. [27].

**Fig 2:**
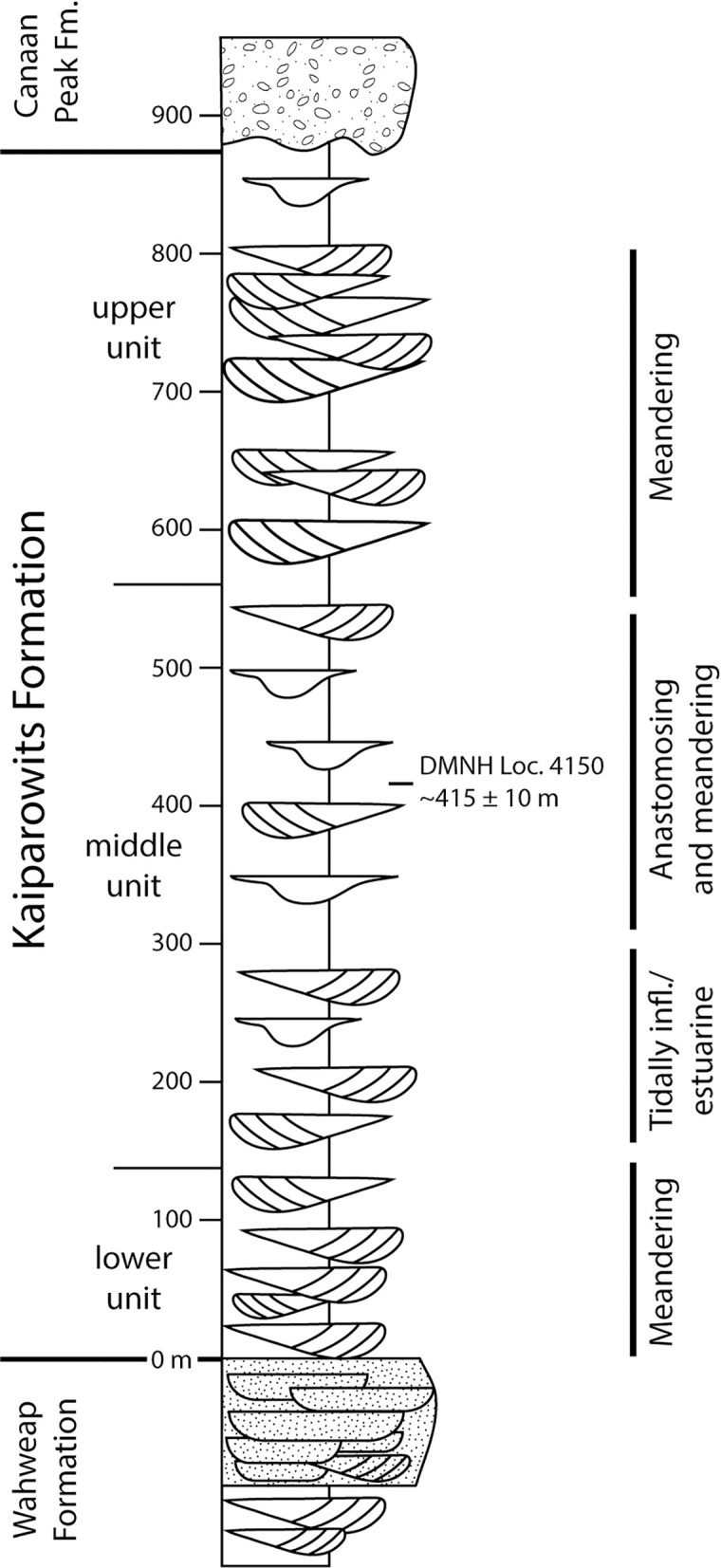
Representative stratigraphic column for the Kaiparowits Formation. Redrawn from Roberts [29] showing major sedimentary modes. The stratigraphic position of DMNH loc. 4150, where the type and referred material for *Catula gettyi* was collected, was located by directly measuring section from the contact with the Wahweap Formation with the assistance of J. Hagadorn and M. Marshall in 2015.

The Kaiparowits Formation is located in south-central Utah, USA, within the newly re-established boundaries of the Grand Staircase–Escalante National Monument (Fig 1). The formation comprises ~1005 m of alternating sandstone and mudstone beds from an array of depositional environments, including channels, lakes, and a variety of floodplain deposits that include crevasse splays, perennial ponds, and oxbow lakes [28–30] (Fig 2). The depositional environment of the Kaiparowits Formation is interpreted as an alluvial to coastal plain, with source material originating from the west along the Sevier orogenic belt and directed to the Western Interior Seaway in the east. ^40^Ar/^39^Ar dating from the Kaiparowits Formation provides an age of ~76.6–74.5 Ma [28,30], placing it within the Campanian Age (83.6 to 72.1 Ma) of the Upper Cretaceous series. Penecontemporaneous formations include the Dinosaur Park Formation in Alberta, Canada, and the Two Medicine and Judith River formations of Montana, USA, among other penecontemporaneous formations from Mexico to Alaska. The paleoenvironment likely was extensively ponded and annually flooded, based on paludal deposits, floral and faunal composition, leaf physiognomy [25,26,29], and isotopic composition of dinosaur teeth [27]. This interpretation, along with temperature estimates from fossil leaves, suggests the climate was humid and subtropical, similar to the present-day Gulf Coast or certain areas of Southeast Asia [27].

The Kaiparowits Formation is informally divided into upper, middle, and lower units, as well as the newly described Upper Valley Member [see 24,30], with the middle unit producing the bulk of floral and faunal specimens. During the past ten years of field exploration, the authors have found and collected more than 100 megafloral localities within the formation. Most of these localities occur in the middle unit, which ranges stratigraphically from about 90–110 m at its base to about 550 m at its uppermost level within the formation [24]. For the middle unit, the majority of megafloral localities are restricted to the stratigraphic interval between about 300 m and 450 m [26]. Based on correlation to the local stratigraphic section for the Fossil Ridge area [24,28], the Lost Valley Locality (DMNH Loc. 4150), is located in the middle unit of the Kaiparowits Formation, approximately 415 ± 10 m above its base. Using a depositional rate of 41 cm/1,000 years [28], which was calculated using ^40^Ar/^39^Ar ages on sanidine crystals from volcanic ash beds, we estimate the age of DMNH Loc. 4150 at 75.6 ± 0.18 Ma. The error of this estimate was propagated from the error associated with the age on the nearest ash bed (Death Ridge Ash [28,29]) and the stratigraphic positions of the ash bed and the fossil locality.

At DMNH Loc. 4150, leaves are preserved as compression-impression fossils in stacked 5–10 cm thick, fine-grained sandstone beds with minor mud partings. The depositional environment is interpreted as a medial to distal crevasse splay resulting from an event or events that infilled a perennial pond or small lake. Using the facies associations of Roberts [28,29], the fossils occur in the FA5 stratum, which consists of minor tabular and lenticular sandstone, immediately above the FA9 stratum, which is carbonaceous mudstone. FA5 is interpreted as forming from crevasse splays and crevasse channels, whereas FA9 is interpreted as forming in swamp and oxbow lake environments [29].

## Materials and methods

### Paleobotany

The plant megafossils from the Lost Valley locality (DMNH loc. 4150) were collected under Bureau of Land Management (BLM) permit UT13-026E-GS by the Denver Museum of Nature & Science (DMNS) for work in the Grand Staircase Escalante National Monument. All permits are on file at DMNS and BLM and may be available upon request. Specific locality information and documentation for DMNH loc. 4150 is available upon request to qualified researchers. The fossil specimens recovered from DMNH loc. 4150 were collected using standard bench-quarrying techniques and we collected all identifiable specimens and did not make a field census because the flora had not been previously sorted into morphotype categories. In the lab, the megafossils were sorted into morphotypes following the concept and procedure described by Johnson [54] and following the morphological terminology of the *Manual of Leaf Architecture* [55]. This method uses the morphological characters of disassociated plant organs, such as leaves, fruits, and stems, to circumscribe discrete operational taxonomic units prior to erection of a formal taxonomy. Each morphotype, based on multiple, well-preserved specimens, closely approximates a biological species. We use the morphotype prefix, KP, to designate the Kaiparowits Formation, followed by a sequential listing of the number of morphotypes in the formation.

The Lost Valley locality (DMNH loc. 4150) contains 4,004 specimens identified to 101 morphotypes. The non-reproductive morphotypes include 8 ferns, 1 lycopod, 1 sphenopsid, 1 gymnosperm, and 59 angiosperms. The reproductive morphotypes include 31 seeds, fruits, and flowers. Of all specimens from this locality, 1,564 (~39%) were assigned to KP89, which is formally described and named below. Specimens of KP89 that were more than a third complete were examined for insect-mediated damage. The majority of specimens were over fifty percent complete. A formal description of this taxon was erected to 1) advance our understanding of the Kaiparowits flora, wherein we begin to circumscribe and name common, well-preserved taxa; 2) provide a foundation for ecological analyses, described below; and 3) allow for comparisons of specialized plant–insect associations between other, described Lauraceae taxa from the Late Cretaceous of North America.

### Nomenclature

The electronic version of this article in Portable Document Format (PDF) in a work with an ISSN or ISBN will represent a published work according to the International Code of Nomenclature for algae, fungi, and plants, and hence the new names contained in the electronic publication of a PLOS ONE article are effectively published under that Code from the electronic edition alone, so there is no longer any need to provide printed copies.

The online version of this work is archived and available from the following digital repositories: PubMed Central, LOCKSS.

### Plant–insect associations

Insect herbivory was documented following a system of identification and classification frequently employed in plant–insect associational studies [56–60]. There are several criteria used to distinguish herbivore induced insect damage from other types of damage, such as physical damage resulting from tears occurring along leaf veins, detritivory involved in the consumption of dead tissue, or taphonomic processes that alter leaf tissue [60]. The first criterion is the presence of reaction tissue. Reaction tissue often occurs as anomalous parenchymatous enlargement, such as callus, that results from hypertrophic (enlarged) or hyperplasic (multiplied) cells produced by the plant along insect damaged areas [61–64]. A second criterion for insect damage is the targeting of a specific host-plant taxon or a particular plant organ that would be attributable to insect-specific patterns of damage. Examples of this type of damage are linear rows of punctures on or along primary veins, or small cusps occurring on the cut edge of a plant tissue [65–68]. A third criterion is a repeated damage pattern based on shape, size, and position of the damage on the plant [69,70]. After herbivore mediated damage was identified on the plant host, it was classified by feeding guild, or functional feeding group, and into specific, diagnosable patterns of insect plant-tissue modification, the damage type [60].

Insect damage was scored following the *Guide to Insect (and other) Damage Types on Compressed Plant Fossils* [60] and subsequent published and unpublished addenda. The damage was initially categorized into one of eight functional feeding groups: 1) hole feeding; 2) margin feeding; 3) skeletonization; 4) surface feeding; 5) oviposition; 6) piercing and sucking; 7) galling; and 8) mining. Oviposition is not herbivory per se but does represent damage to the foliar tissue of plants that elicits defense responses and has a persistent fossil record [71–75]. Similarly, galls may be created by insects, mites, nematodes, fungi, bacteria, or viruses [76,77]. Galls may be formed in conjunction with piercing and sucking and may or may not be associated with herbivory [76,77], but are herein categorized as insect damage. Discrete, diagnosable damage types were documented within each functional feeding group and assigned a damage type (DT) number. Damage types are rated for host specificity: 1 or generalized, similar to polyphagy for modern insects; 2 or intermediate, similar to oligophagy; and 3 or specialized, similar to monophagy [60]. Damage types are rated for host specificity: 1 or generalized (polyphagous), 2 or intermediate (oligophagous), and 3 or specialized (monophagous) [60]. The convergence of herbivore mouthparts and feeding behaviors make genus or species level identifications of the insect culprit rare; some margin feeding, most leaf mines, galls, and many scale-insect feeding marks are traceable to lineages with living representatives [78–82].

Herbivory data collected from insect damaged leaves includes both qualitative and quantitative assessments. The qualitative data, outlined above, determine the overall insect feeding guilds on a particular host-plant taxon, the richness of damage types, host specificities, and occasionally the identity of the phytophagous insect responsible for the damage [60]. Quantitative data collection consisted of four basic metrics: 1) the proportion of damaged leaves, 2) the richness of damage types, 3) the abundance of damage types, and 4) the percent of surface area herbivorized by insects (herbivory index), which measures the intensity of herbivory. For calculating the surface area of leaf tissue herbivorized by insects, a subset of 156 specimens of the new taxon (10% of total specimens) was randomly selected using the random number generator package “Rando” for R statistical software [83].

**Fig 3:**
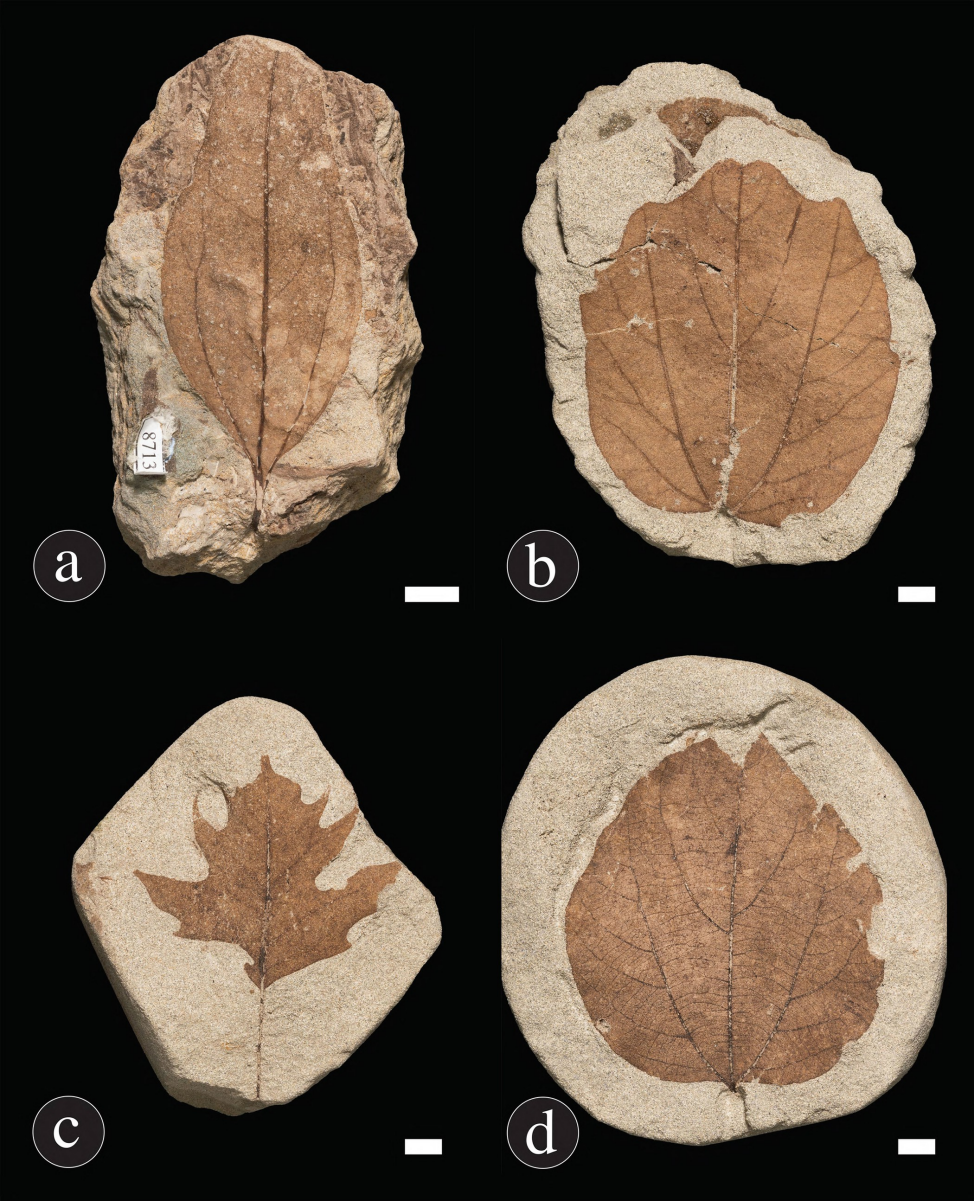
Photographs of Hell Creek taxa. (A) *Marmarthia pearsonii*, (B) *M*. *trivialis* (C) “*Artocarpus*” *lessigiana*, and (D) “*Ficus*” *planicostata*. Black scale bars = 10 mm; white scale bar = 10 mm. Photographs by R. Wicker, DMNS.

Four additional taxa attributed to the family Lauraceae from channel deposits the Hell Creek Formation by Johnson [17,84,85] were included in the final analysis (Figs 3 and S1): *Marmarthia pearsonii* (DMNH loc. 900; 34.4% of the flora at that locality), *Marmarthia trivialis* (DMNH loc. 428; 6.9% of the flora at that locality), “*Artocarpus*” *lessigiana* (DMNH loc. 428; 3.3% of the flora at that locality), and “*Ficus*” *planicostata* (DMNH loc. 428; 2.4% of the flora at that locality) [84]. Each Hell Creek Formation taxon with a sample size of at least 20 specimens from a single locality was analyzed for insect damage as outlined above. Comparisons were made to these four taxa to provide context for the level of herbivory on the Kaiparowits laurel described herein, as well as to determine if there are any specialized damage types that persist throughout the Late Cretaceous. These taxa, which were previously analyzed for insect herbivory by Labandeira et al. [86,87], were selected based on spatiotemporal proximity, as these specimens are from North Dakota, USA, and are Maastrichtian in age. Although there are differences in the sampling intensities and local abundances among the five taxa, wherein the Hell Creek taxa ranged from 23 to 167 specimens, we randomly sampled Kaiparowits specimens (156 leaves) to fall within that range.

Detailed photographs were taken using a Canon EOS 50D camera with a Canon EF-D 60mm f/2.8 macro lens and microphotographic images were taken using an Olympus DP25 camera attached to an Olympus SZX12 microscope. Digital images were processed using Adobe Photoshop CC® (2017.01) and Zerene Stacker® software. Surface area for all five taxa in this study was measured using Adobe Illustrator Draw^©^ for iPad Pro and ImageJ [88]. Plates were created using Adobe InDesign CC® (2017.1).

Sample-based rarefaction was calculated for the damage type richness and sampled surface areas of the plant hosts, as it allows for comparisons of insect damage richness between taxa. The rarefaction analysis by total sampled surface area was used instead of number of specimens because this standardizes differences in leaf size and leaf completeness between species. A rarefaction analysis and resulting curves were created using code developed by S. Schachat [89,90] for R statistical software [83]. Rarefaction curves were bootstrapped 5,000 times to generate 84% confidence intervals. The herbivory index was calculated for the Hell Creek and Kaiparowits taxa and 95% confidence intervals from bootstraps of 10,000 iterations.

Nonmetric multidimensional scaling (NMDS) ordinations, which used a Bray-Curtis dissimilarity matrix, were produced via the metaMDS function of the vegan package, in R version 3.1.2, also used in previous studies [91,92]. NMDS plots represent the positions of data in multidimensional space that allow for visual comparisons between plant hosts. Because null values are extremely common in plant–insect associational datasets, this dissimilarity matrix and ordination method were selected because they are not affected by null values, as opposed to methods such as principal component analysis (PCA) [93]. The NMDS plot was produced with the R package ggplot2 [83]. To standardize for sampling effort and to quantify uncertainty, each of the Kaiparowits and Hell Creek taxa were subsampled 500 times to a given amount of surface area. This process was repeated nine times, setting the seed in R from 1 to 9. For the first series of NMDS plots, all five taxa were subsampled to 850 cm^2^ of surface area; “*A*.” *lessigiana* is represented by 884.85cm^2^ of surface area. For the second series of NMDS plots, “*A*.” *lessigiana* was removed from the dataset and the remaining four taxa, which are represented by between 1420.36 and 1707.48 cm^2^ of surface area, were subsampled to 1400 cm^2^ of surface area. Ellipses contain 84% of points closest to the centroid of each taxon and represent 84% confidence intervals.

## Results

### Leaf morphology and systematics

Leaves of the new fossil taxon are herein described based on the suite of 1,564 specimens from the Lost Valley locality (DMNH loc. 4150) in the Kaiparowits Formation, Utah, USA.

### Systematics

Order: Laurales (Juss. ex Bercht. & Presl, 1820) [94]

Family: Lauraceae (Jussieu, 1789 *nom*. *cons*.) [95]

*Catula* Maccracken, Miller, Johnson, Sertich, Labandeira, gen. nov.

#### Generic diagnosis

Leaves simple; when attached, distichous, exhibiting opposite or slightly subopposite arrangement and axillary buds. Lamina nearly always slightly asymmetrical in the apex, middle, and base of the leaf. Leaf margin entire and unlobed. A fimbrial vein observable in well-prserved specimens. Leaf apex typically acute and often exhibiting a mucronate termination. Leaf base typically acute and markedly decurrent, with laminar tissue extending down the petiole. Primary venation pinnate. Secondary venation simple brochidodromous; associated with simple agrophic veins. Secondary veins basally crowded to form 1 or 2 pairs of acute basal secondary veins. Tertiary venation opposite percurrent. Exterior tertiary veins and ultimate observable venation looped. Higher order venation relatively disorganized and the leaf rank is 2r. Quaternary vein fabric regular to irregular reticulate and quinternary vein fabric irregular reticulate.

#### Derivation of the generic name

From the masculine noun *catulus*, a Classical Latin noun meaning a young animal (Pliny), especially a young dog, puppy or whelp (Cicero, Lucretius, Vergil). The genus epithet is, in part, named for Mike Getty’s dog, Javelina, who also answers to the name, Puppy. The diminutive suffix–*ulus*/-*a* also describes the small size of many of the leaves in this genus.

#### Discussion

In establishing the new genus, *Catula*, we examined morphologically similar fossil genera, as well as extant genera, that would allow for the classification of the vegetative (foliar) characters of the new plant taxon from the Kaiparowits Formation that we describe below. Based on the combination of simple leaves, distichous and opposite or slightly subopposite leaf arrangement with axillary buds, entire margins, pinnate primary venation, simple brochidodromous secondary venation, and a markedly decurrent base with 1 or 2 pairs of well-developed acute basal secondary veins, we hypothesize that the newly diagnosed genus, *Catula*, belongs to Lauraceae.

In addition to these foliar characters, and the new species described in the genus below, the laminar shape of *Catula* is primarily ovate, occasionally elliptic, or rarely obovate. The leaves are also variable in length, width, and size, which leads to additional variation in shape. The opposite percurrent tertiary venation can also be variable across individual specimens and between specimens, as the venation can have a variety of courses and an inconsistent angle relative to the primary vein. Considering this suite of characters, plus those listed in the diagnosis, we found that Early Cretaceous through Paleogene fossil species assigned to the extant genera *Cinnamomum*, *Cocculus*, *Laurus*, *Malapoenna*, *Nectandra*, *Persea*, and *Sassafras* in the Lauraceae; *Paliurus* in the Rhamnaceae; *Ficus* in the Moraceae; *Populus* in the Salicaceae; and *Pieris* in the Ericaceae resembled our new taxon. Arguably, most, and perhaps all of these generic names are misapplied to these taxa as is common in historical paleobotanical literature. While the fossil species that exhibit the most similar leaf morphology and vein architecture to our new taxon in *Catula* are distinguished (see below for discussion of “*Cinnamomum”* spp.), our new taxon is based only on sterile material, as no cuticular or fertile material has been recovered or associated with these leaf fossils. As a result, we elect not to assign it to an extant genus where attribution necessarily includes reproductive characters. This argument is bolstered by the fact that the fossils we describe are approximately 75 million years old and attribution to a living genus would require extraordinary morphological evidence.

In Lauraceae, *Catula* exhibits leaf attachment, leaf shape, and primary, secondary, and tertiary vein characters consistent with species in extant *Cinnamomum* [96] or the *Cinnamomum–Ocotea* clade [97]. Within this clade, *Catula* is more similar to venation patterns present in *Cinnamomum* [96] though it can be difficult to differentiate species of *Cinnamomum* and *Ocotea* simply on vegetative characters. Until recently [98], Old and New World species in *Ocotea* have been considered by most workers to form a polyphyletic or, at least, paraphyletic clade [99,100]. New work has led to well-supported clades, including proposing some outlying Old World *Ocotea* species be reclassified in the new genus *Kuloa* [101]. This new classification is relevant in that a key diagnostic feature of *Kuloa* is (sub)opposite leaf attachment, which occurs in *Kuloa*, *Cinnamomum*, and *Catula*, but not in the vast majority of *Ocotea* with the exception of rare, unresolved examples [101]. For the species of *Ocotea* reclassified in *Kuloa*, they are distinct from *Catula* in having markedly different second and third order venation characteristics. As a result, we argue that *Catula* is most closely allied with extant *Cinnamomum*.

Several workers have argued that *Cinnamomum* exhibits two characteristic primary venation patterns [ex. 102–104]: acrodromous venation typified by *Cinnamomum verum* (cinnamon), and pinnate venation typified by *Cinnamomum camphora* (camphor). We examined herbarium sheets of 133 species of *Cinnamomum* (~38–53% percent of the 250 [105] to 350 [2] species) in the Smithsonian’s National Museum of Natural History virtual botany collections and the New Botanical Gardens Steere Herbarium C.V. Starr Virtual Herbarium and found additional support for these venation patterns, plus a third pattern. These patterns are: 1) an acrodromous primary venation pattern with weakly expressed brochidodromous to eucamptodromous secondary veins and prominent, usually well-organized opposite percurrent tertiary veins. This venation pattern would be considered “triplinerved.” 2) A pinnate primary venation pattern with prominent basal secondary veins that have an acrodromous or brochidodromous course. In the distal portions of these leaves, additional well-defined, brochidodromous secondary veins occur. Tertiary veins in this category are typically alternate percurrent to mixed opposite and alternate percurrent This venation pattern may be considered “triplinerved.” And 3) a pinnate venation pattern that does not have prominent basal secondary veins. Secondary veins in this category are typically of the same gauge or are reduced in gauge uniformly from the base of the leaf to the apex. Tertiary veins in this category range considerably in course and organization. This venation pattern would be considered “penninerved.” Considering these venation patterns, *Catula* appears more closely allied with the “intermediate” category exhibiting pinnate venation with prominent basal secondary veins.

The morphology of our taxon also falls within generalized fossil leaf-morphotype “Cinnamomophyll A” in the scheme described by Crabtree [106] and exemplified by *"Cinnamomum" sezannense* (Lesquereux [107]) from the Dakota Formation. While not a recognized name, Cinnamomophyll highlights the suite of characters exhibited by our taxon and allies it with Cretaceous “lauroid” fossil taxa. Among fossil form genera, we found five that might accommodate our new taxon. These are: *Laurophyllites* Weyland & Kilpper [108] for penninerved leaves attributable to the Lauraceae; and *Cinnamomoides* Berry [Seward] [109], *Cinnamomophyllum* Kräusel and Weyland [110] later synonymized with *Daphnogene* (Kvaček and Knobloch, [111]); *Daphnogene* (Unger [Brongniart], [112]), and *Laurophyllum* Goeppert [113], for triplinerved leaves attributable to the Lauraceae [see 6]. While pinnate, *Catula* more closely resembles the triplinerved form genera (*Laurophyllites*).

We have elected to erect *Catula* because we have found that the existing similar form genera in Lauraceae available to us are imperfect categories incorporating taxa of global distribution ranging from Early Cretaceous through Neogene time, limiting, or even eliminating, their taxonomic, phylogenetic, and temporal utility. An example of problems arising from analyses of form genera includes a recent study on the long-term plasticity of *Daphnogene cinnamomifolia* in which the authors acknowledged the likelihood that this taxon represents multiple Cenozoic taxa [114]. Furthermore, specimens of *Laurophyllum* span a time interval from at least the Late Cretaceous (Campanian, 83.6–72.1 Ma) to the Pleistocene (2.58–0.012 Ma) and four continents, a significant portion of the 108-million-year history and geographic distribution of the Lauraceae [115]. Beyond this issue, these form genera are considered polyphyletic and based on only a few, simple venation characters [6]. Where possible, workers should define generic boundaries more precisely, as we are able to do in the case of *Catula*. Assigning species in well-described and diagnosed genera with clear stratigraphic and geographic limitations preserves taxonomic information and allows for phylogenetic biogeographic inferences leading to their utility in meta-analyses, like the suite of plant–insect associations we present herein, or future, regional studies of the evolution of Lauraceae in the Western Interior of North America. In our search of the literature, and with these arguments in mind, we did not find a suitable fossil (form) genus for the new taxon described below and thus we erect *Catula* and hypothesize that it belongs in the Lauraceae.

*Catula gettyi* Maccracken, Miller, Johnson, Sertich, Labandeira, sp. nov.

**Fig 4:**
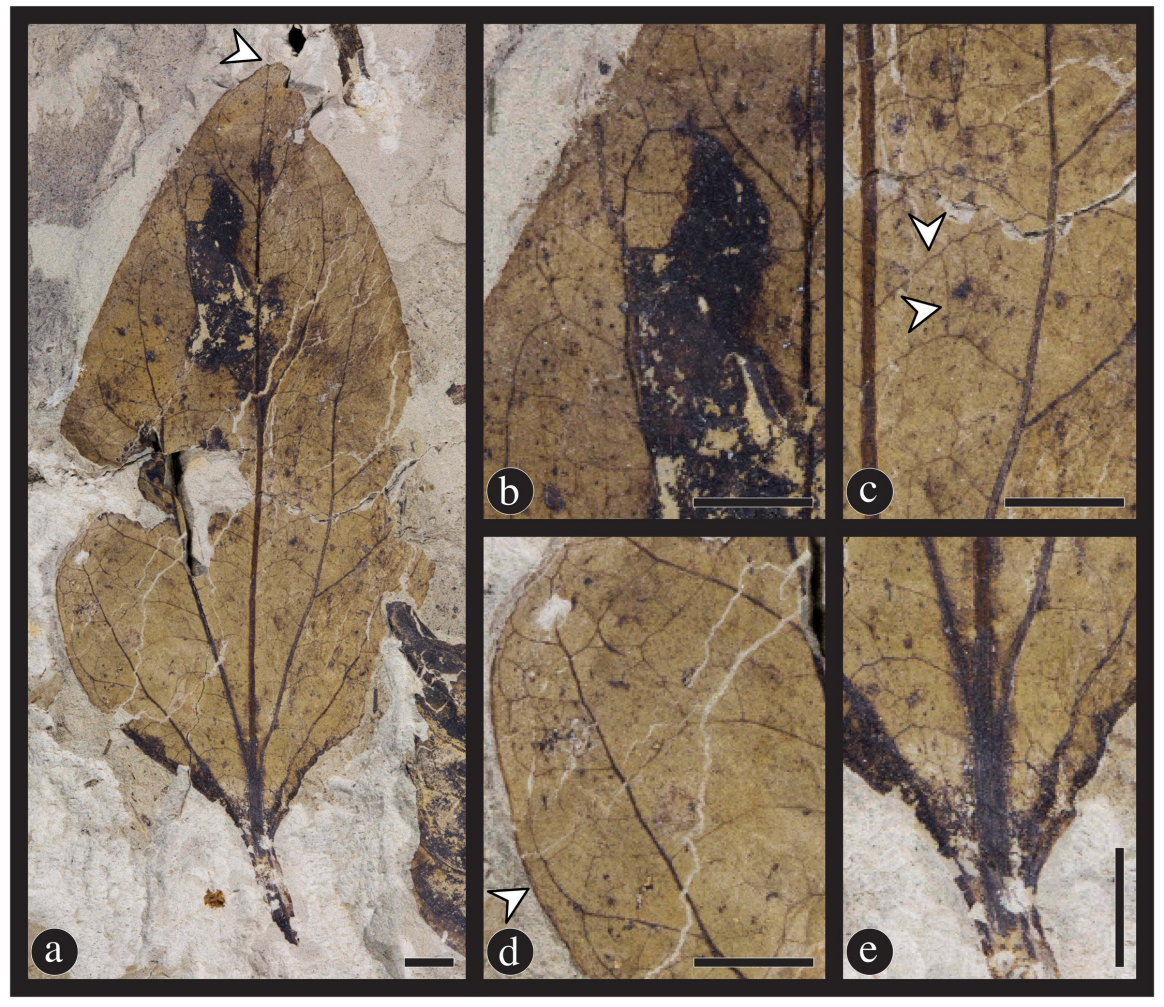
Overall and detail images of the holotype specimen (DMNH 54376, DMNH loc. 4150) of *Catula gettyi* Maccracken et al., gen. et sp. nov. (A) Complete leaf. Arrow indicates mucronate apex. (B) Detail showing upper left section of the leaf with looping and simple brochidodromous secondary venation. The dark area on the leaf is a blotch mine. The primary vein of the leaf parallels the right side of the figure. (C) Detail showing a medial section on the right side of the leaf, with the primary vein on the left side of the figure. Note the epimedial tertiary veins with variable course (upper arrow), and the irregular to regular reticular quaternary venation (lower arrow). (D) Detail showing lower left section of the leaf with simple agrophic veins, looping ultimate marginal venation, and a fimbrial vein indicated by arrow. (E) Detail showing the base of the leaf with 2 pairs of acute basal secondary veins and laminar tissue extending down the petiole. Note the decrease in the primary vein width with the departure of the secondary veins. All scale bars = 0.5 cm.

**Fig 5:**
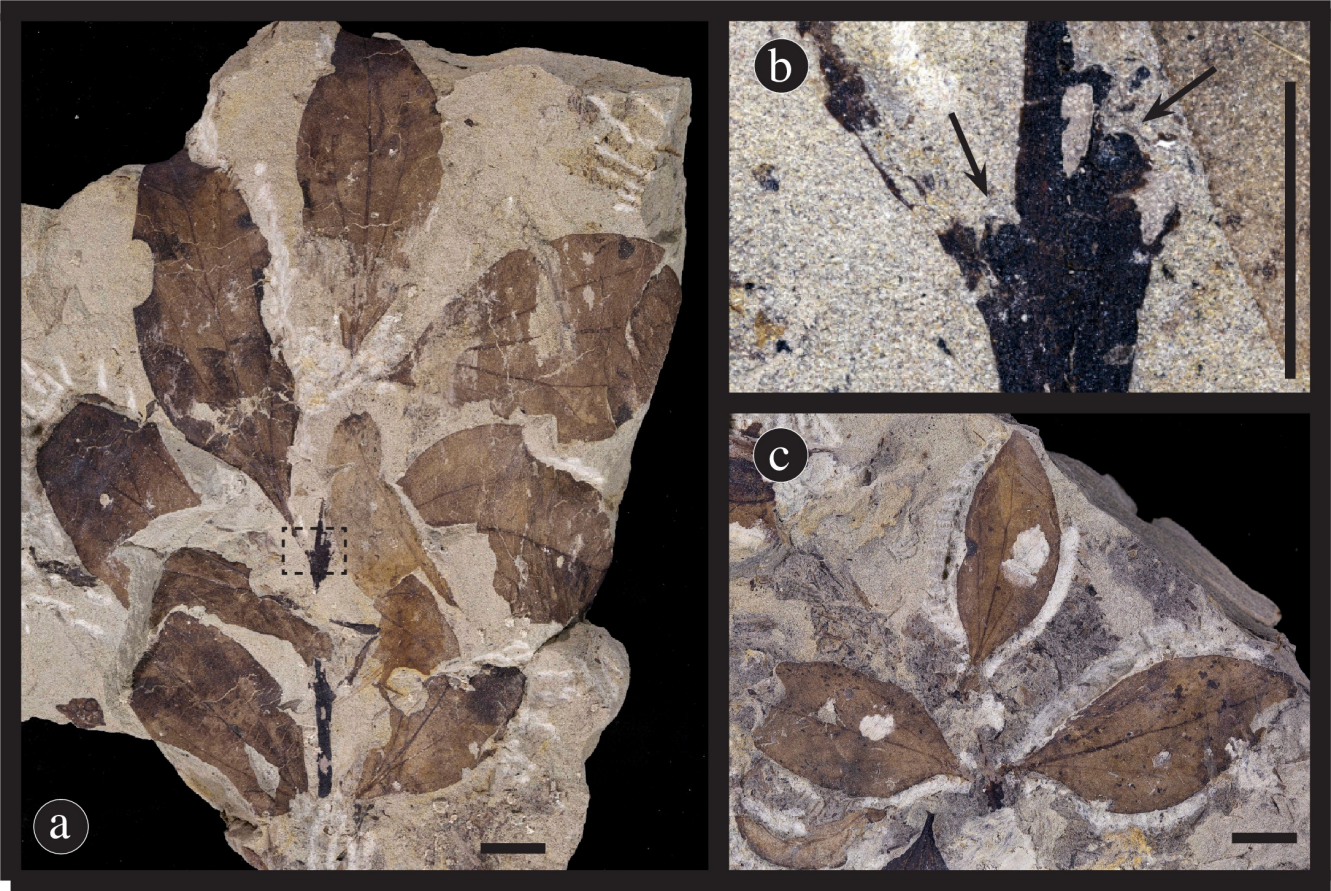
Paratype specimens of *Catula gettyi*. (A) Attached leaves of *C*. *gettyi* on a stem showing opposite leaf attachment, probable distichous arrangement, and an odd-pinnate leaf terminus (DMNH 54378). Scale bar = 1 cm. (B) Enlarge section of dashed inset box in Fig 4 a showing leaf attachment (DMNH 54378). Arrows highlight axillary buds. Scale bar = 0.5 cm. (C) Attached leaves of *C*. *gettyi* on a stem showing opposite leaf attachment and an odd-pinnate leaf terminus (DMNH 54377). Scale bar = 1 cm.

**Fig 6:**
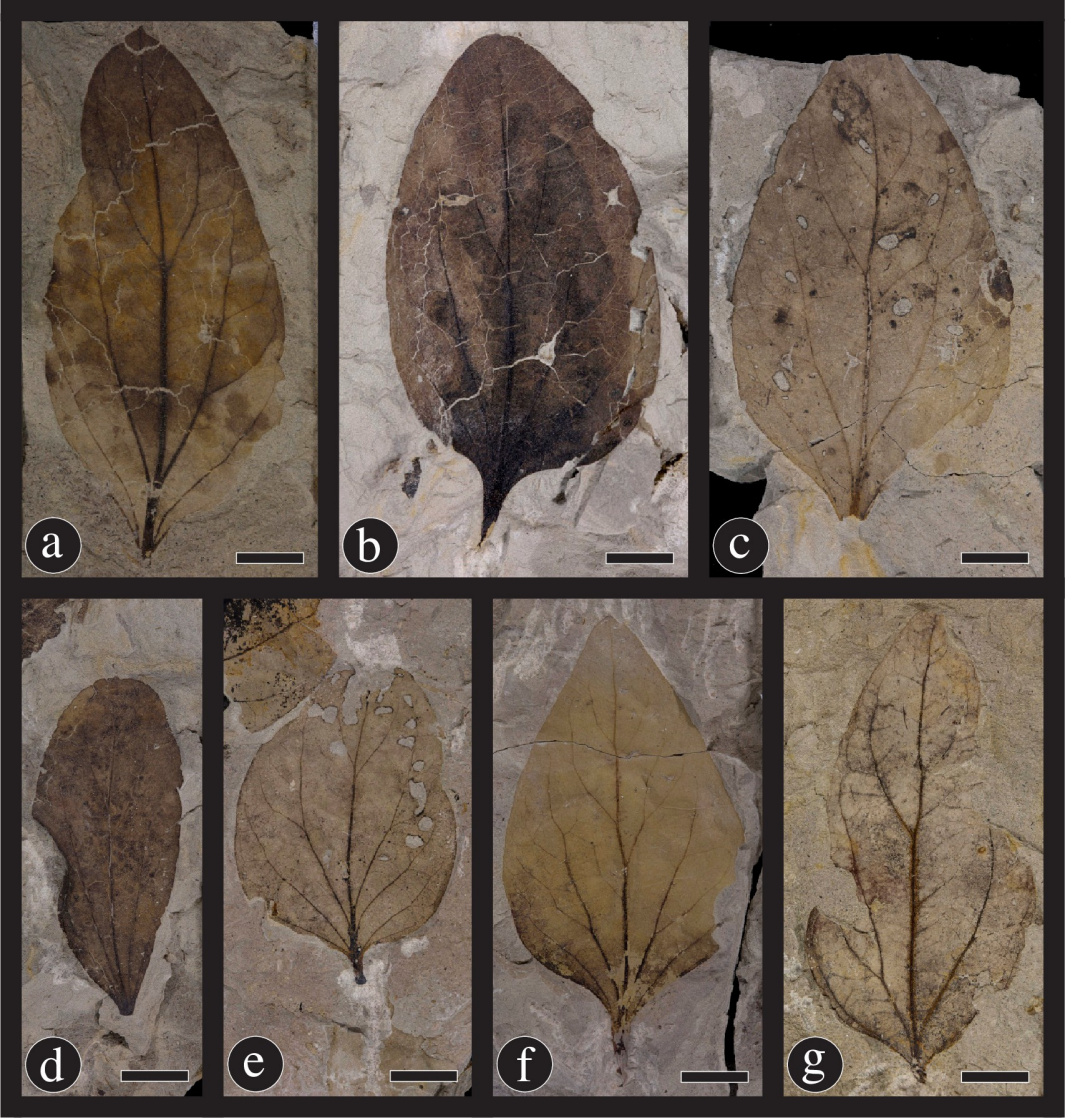
Paratypes of *Catula gettyi*. These specimens show the range of leaf architecture exhibited by this taxon. (a) DMNH 41570. (b) DMNH 54371. (c) DMNH 54379. (d) DMNH 54370. (e) DMNH 41584. (f) DMNH 41567. (g) DMNH 54380. Scale bars = 1.0 cm.

Figs 4A–4E, 5A–5C and 6A–6G.

#### Specific diagnosis

Same as the generic diagnosis by monotypy.

#### Holotype

Designated here: DMNH 54376 (Fig 4A–4E).

#### Paratypes

Designated here: DMNH 54378 (Fig 5A and 5B–stem with many attached leaves), DMNH 54377 (Fig 5C–stem with 3 attached leaves), DMNH 41570 (Fig 6A ‒ single leaf), DMNH 54371 (Fig 6B ‒ single leaf) DMNH 54379 (Fig 6C ‒ single leaf) DMNH 54370 (Fig 6D ‒ single leaf) DMNH 41584 (Fig 6E ‒ single leaf) DMNH 41567 (Fig 6F ‒ single leaf) DMNH 54380 (Fig 6G ‒ single leaf).

#### Other figured specimens

Figs 8‒15

#### Derivation of the specific epithet

In recognition of Michael A. Getty for his nearly two decades of incredible support and leadership in uncovering the paleontological treasures of Grand Staircase-Escalante National Monument.

#### Source, age, and stratum

*Catula gettyi* is found at numerous localities throughout middle unit of the Kaiparowits Formation spanning perhaps as much as 1 myr. All *C*. *gettyi* specimens are housed at the Denver Museum of Nature & Science. Precise GPS locality information is available upon request.

#### Description

*Catula gettyi* occurs mostly as isolated leaves, while a few specimens show leaves attached to stems. Leaf attachment petiolate; leaf arrangement opposite to subopposite, appearing distichous; even and odd pinnate terminus on the stem; leaf organization simple. Auxiliary buds present in leaf axils. Petiole twisted, sometimes flanked with a thin wing of laminar tissue from the blade; petiole base slightly swollen. Blade attachment marginal. Laminar size notophyll, rarely nanophyll to mesophyll; laminar length variable but generally 4 to 8 cm; laminar width variable but generally 2.5 to 4.5 cm; laminar length to width ratio generally 1:1.0 to 3:1; laminar shape ovate or occasionally elliptic, or rarely obovate; medial symmetry slightly asymmetrical, rarely symmetrical. Laminar base slightly asymmetrical, rarely symmetrical, occasionally with a slight asymmetrical basal insertion; base angle acute; base shape decurrent. Laminar apex angle acute, rarely obtuse; apex shape straight to acuminate; laminar apex with a mucronate termination in some specimens, otherwise appearing slightly retuse. Leaf margin entire, unlobed; laminar edge appearing thickened or with an observable fimbrial vein of tertiary or higher order; laminar surface texture appearing smooth. Primary venation pinnate; thickness of primary vein up to ~ 1.3mm; course of primary vein approximately straight; primary vein markedly decreases in width after giving rise to major secondary veins, particularly near the base of the leaf. Secondary vein organization simple brochidodromous; agrophic veins present, simple; 1‒5 or rarely 7 basal veins including both primary and secondary veins; naked basal veins present, of secondary or tertiary vein order; spacing of secondary veins on primary vein decreases proximal to the leaf base, forming 1 or 2 pairs of acute basal secondary veins; typically 4 pairs of secondary veins; angle of secondary vein departure from primary vein acute; secondary vein course generally arching towards leaf apex, decurrent on the primary vein, course deflected at the origin of minor secondary veins; minor secondary vein course simple brochidodromous; interior secondary veins absent; intersecondary veins absent. Intercostal tertiary vein organization opposite percurrent and sinuous to convex; tertiary vein course angle with respect to the primary vein acute; tertiary vein angle variability with respect to the primary vein inconsistent. Epimedial tertiary veins alternate percurrent; proximal course acute to the midvein, distal course basiflexed. Exterior tertiary course looped. Quaternary vein fabric regular to irregular reticulate. Quinternary vein fabric irregular reticulate. Higher order venation obscured. Marginal ultimate venation appearing looped. No cuticular or fertile material recovered or associated with these leaf fossils.

#### Discussion

We compared the *Catula gettyi* specimens to Early Cretaceous through Eocene leaves in North America [105,109,116–120]. Despite the abundance of fossil “lauroid” leaves in the literature, we found few favorable matches to *C*. *gettyi*. Of the fossils most similar to *C*. *gettyi* were fossils assigned to the extant genus *Cinnamomum* in the Lauraceae. In particular, “*Cinnamomum” newberryi* Berry [109] and “*Cinnamomum” newberryi ellipticum* Berry [109] from the Maastrichtian Ripley Formation in Texas are similar in many aspects to *C*. *gettyi* but differ by having a narrower leaf shape, prominent agrophic veins and better organized opposite percurrent epimedial tertiaries with more or less straight courses. *“Cinnamomum” affine* Lesquereux in Knowlton [96] from the Campanian Mesaverde Formation, and *“C*.*” affine* Lesquereux [97] from the Maastrichtian Laramie Formation share characters with *C*. *gettyi* based on leaf shape and two pairs of acute, basal, secondary veins, but differ by exhibiting better organized opposite percurrent epimedial tertiaries with straight courses. The lauraceous taxon *Marmarthia pearsonii* Johnson [17] from the Maastrichtian Hell Creek Formation resembles *C*. *gettyi*, particularly from the perspective of higher order venation and overall low leaf rank. However, *M*. *pearsonii* differs from *C*. *gettyi* by having primary venation that is basal acrodromous as opposed to pinnate, only one pair of prominent basal veins (primary or secondary), more prominent epimedial tertiary veins, and a naked base. Finally, *“Cinnamomum” linifolium* Knowlton [96] from the Paleocene Raton Formation bears resemblance in overall shape and primary and secondary venation to *C*. *gettyi*, but the specimens are too poorly preserved for taxonomic comparison outside the formation.

The 15 most comparable taxa that we observed are listed in S1 Table. While *C*. *gettyi* compares favorably to these taxa, there are notable differences in vein organization. Collectively, these differences show that the veins of *C*. *gettyi* are less organized, leading to an overall lower leaf rank [119], than any taxa we observed in *Cinnamomum*. Given the poorly organized leaf venation of *C*. *gettyi*, and without floral, epidermal, and petiolar/laminar (e.g. domatia) characters [102] to assign the new taxon in *Cinnamomum*, we have elected to erect the new genus and species *Catula gettyi*. Furthermore, based on molecular evidence, *Cinnamomum* appears to have an early Eocene Laurasian origin suggesting Cretaceous attributions to the genus are incorrect [121].

*Catula gettyi* represents the single most abundant leaf megafossil found in the Kaiparowits Formation based on the current collection at the Denver Museum of Nature & Science. In many proximal crevasse-splay floras from the formation, *C*. *gettyi* is the dominant taxon. While a comprehensive analysis of splay, channel, and pond floras in the formation has yet to be completed, it nonetheless appears that *C*. *gettyi* tracks stream margins, and thus disturbed, environments in the formation. Based on the exceptional preservation of this taxon that often obscures higher order venation when compared to the majority of other leaves preserved in DMNH loc. 4150, *Catula gettyi* likely had a robust leaf that may have been evergreen.

### Plant–insect associations on *Catula gettyi*

**Fig 7:**
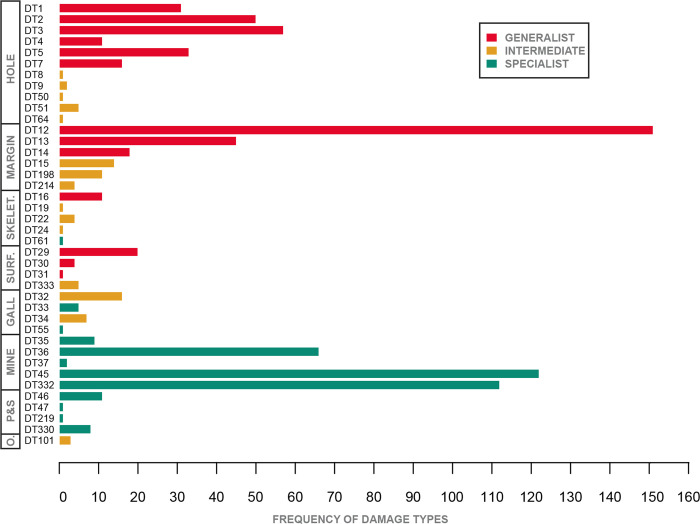
Histogram of all damage types encountered on *Catula gettyi* by functional feeding group. Red bars = generalist host specificity, gold bars = intermediate host specificity, and green bars = specialized host specificity.

We identified 40 distinct patterns of herbivore damage (damage types, or DTs) on *Catula gettyi* leaves at Lost Valley (DMNH loc. 4150) (Table 1; Fig 7). A total of 863 damage-type occurrences were present and the percentage of *C*. *gettyi* leaves with at least one type of insect damage at this locality was 38.75% (606 damaged specimens, including some specimens with multiple damage types). For 156 randomly selected *C*. *gettyi* leaves, the herbivory index, or the percentage of herbivorized surface area is 2.102% (Table 1). The 95% confidence interval ranges from 1.36% to 3.03% (S2 Fig).

**Table 1:** Richness of damage types by functional feeding group and host plant specialization on *Catula gettyi*.

*Host plant specificity*	Damage Types								
	HF	MF	SK	SF	PS	OV	MI	GA	Total
Generalist	6	3	1	3	0	0	0	0	13
Intermediate	5	3	3	1	0	1	0	2	15
Specialist	0	0	1	0	4	0	5	2	12
Total	11	6	5	4	4	1	5	4	40

Abbreviations are: HF, hole feeding; MF, margin feeding; SK, skeletonization; SF, surface feeding; PS, piercing and sucking; OV, oviposition; MI, mining; and GA, galling.

The ectophytic functional feeding groups of hole feeding, margin feeding, skeletonization, and surface feeding, were the most diverse and abundant modes of feeding on *C*. *gettyi*, with a total of 26 distinct damage types and 498 occurrences. There were 14 damage types and 365 damage-type occurrences of endophytic functional feeding groups (i.e. piercing and sucking, oviposition, mining, and galling) on *C*. *gettyi*. In addition, the presence of fungal necroses is commonly associated with insect herbivory; however, no clear insect-mediated fungal damage was encountered on *C*. *gettyi*, as it was difficult to distinguish fungus from discoloration associated with decay and burial. Fungus was most commonly found on poorly preserved and physically damaged specimens, which indicates that fungal attack occurred post-senescence.

#### Hole feeding

**Fig 8:**
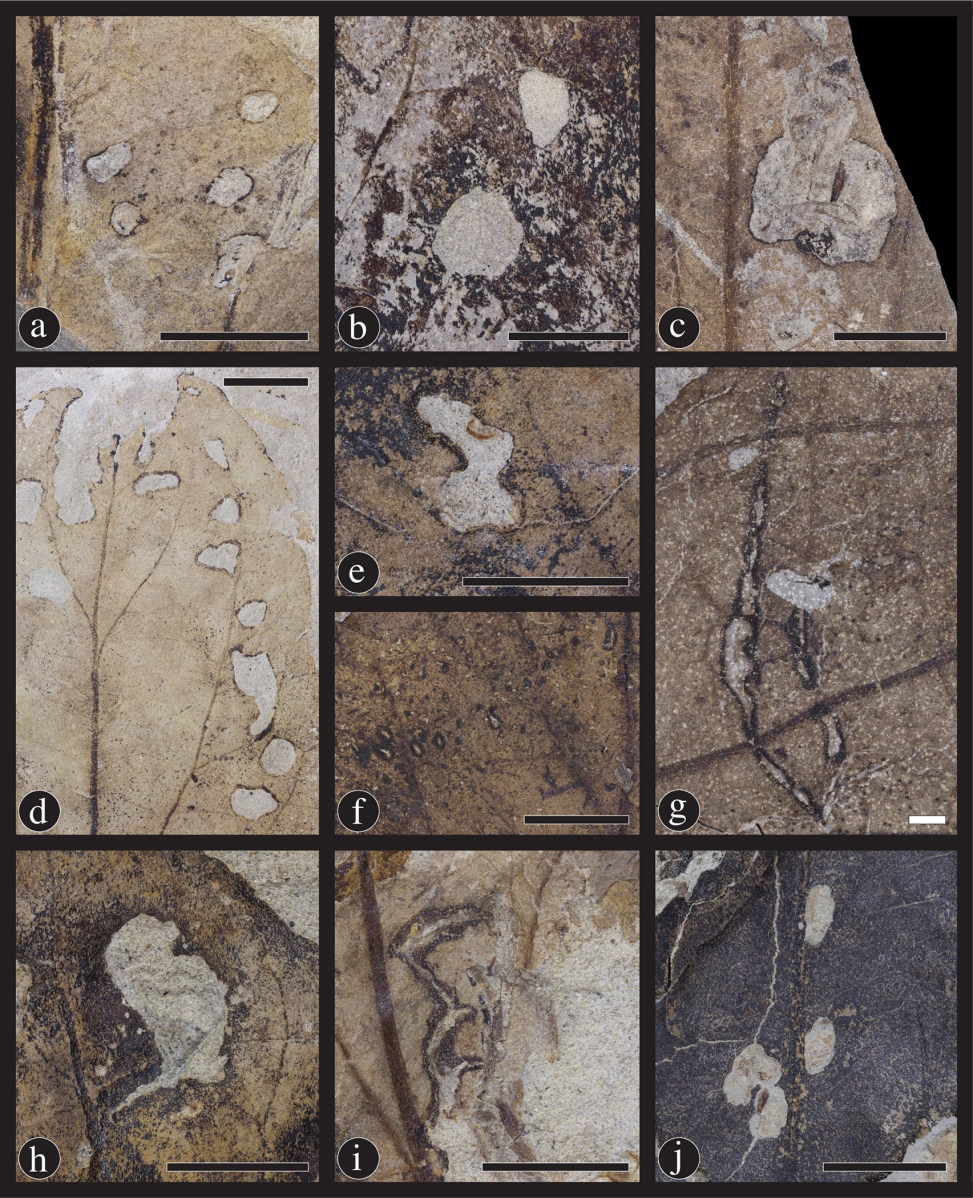
Eleven hole-feeding damage types found on *Catula gettyi* produced by mandibulate insects. (A) DT1; Circular holes under 1mm in diameter (DMNH 41564); (B) DT2; Circular holes between 1 mm and 5 mm in diameter (DMNH 41580); (C) DT4; Circular holes greater than 5 mm in diameter (DMNH 41596); (E) DT3; Polylobate holes between 1 mm and 5 mm in diameter (DMNH 41576); and (H) DT5; Large polylobate holes over 5 mm in diameter (DMNH 41583). Less common hole-feeding types consist of (G) DT7 & DT8 (DMNH 41590); Rectilinear holes and slot feeding, respectively (DMNH 41590); (F) DT9; Scattered, comma-shaped holes (DMNH 39733); (D) DT64; Holes located along the margin of the leaf (DMNH 41584); (I) DT51; Overlapping slot feeding holes (DMNH 41571); and (J) DT50; A series of holes associated with a primary vein (DMNH 41574). Black scale bars = 5 mm; white scale bars = 1 mm.

Hole feeding is the consumption of a leaf, which includes the entire thickness of the lamina and does not reach the leaf margin. Hole feeding on *Catula gettyi* is common and diverse, with eleven damage types and 207 occurrences of DT1, DT2, DT3, DT4, DT5, DT7, DT8, DT9, DT51 and DT64 (Fig 8). Circular hole feeding damage types include holes below 1 mm in diameter (DT1) (Fig 8A), holes between 1 mm and 5 mm in diameter (DT2) (Fig 8B), holes above 5 mm in diameter (DT4) (Fig 8C), and a series of three or more circular holes along the leaf margin (DT64) (Fig 8D). Polylobate hole feeding includes holes between 1 mm and 5 mm (DT3) (Fig 8E), and holes over 5 mm in diameter (DT5) (Fig 8H). DT7 are rectilinear feeding (Fig 8G); DT51 consists of overlapping slot feeding (Fig 8I); and DT9 are elliptical to comma-shaped holes scattered across the leaf surface (Fig 8F). Finally, DT50 is a linear series of holes alongside a primary or secondary vein that occur on one side or on alternating sides (Fig 8J).

#### Margin feeding

**Fig 9:**
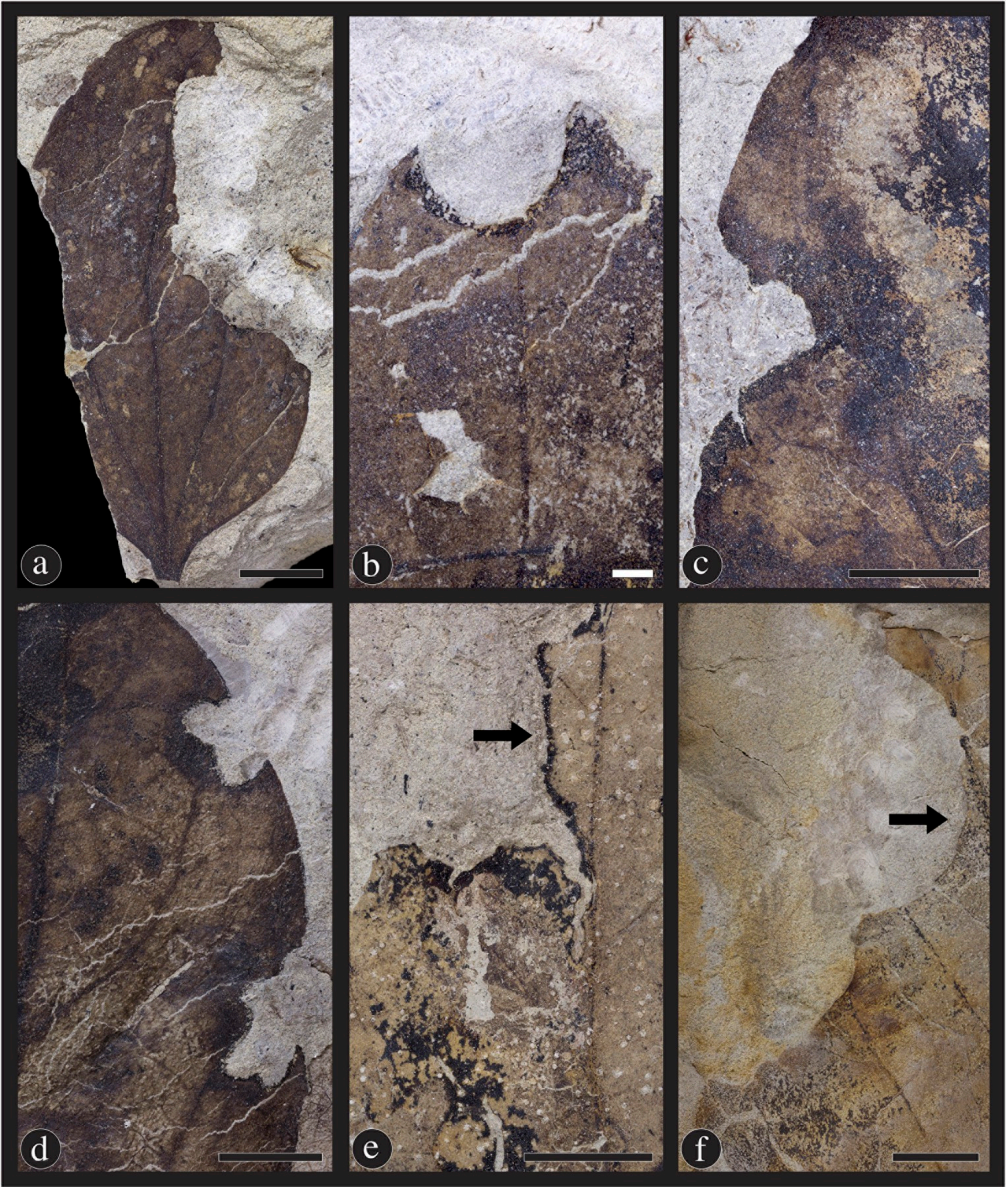
Margin feeding damage types found on *Catula gettyi* at the Lost Valley Locality. (A) DT14; Excision of the leaf to the primary vein (DMNH 41575); (B) DT13; The removal of the apex of leaf by the insect (DMNH 41578); (C) DT12; A common semi-circular excision of the leaf margin (DMNH 41579); (D) DT15; An excision that expands medially (DMNH 41585); (E) DT198; A deep, narrow excision with broad reaction tissue surrounding the herbivorized section (DMNH 41595); and (F) DT214; Multiple, connected excisions along the leaf margin (DMNH 41586). Black scale bars = 5 mm; white scale bars = 1 mm; arrows denote reaction tissues.

Margin feeding is the consumption of the entire thickness of the lamina along the leaf edge by a chewing phytophagous insect. The six distinct margin-feeding damage types on *C*. *gettyi* specimens are DT12, DT13, DT14, DT15, DT198 and DT214 (Fig 9). DT12 is the cuspate and moderately incised, and isolated removal of tissue at the leaf margin; it represents the most common damage type on *C*. *getty*i (Fig 9C). DT13 is the removal of tissue at the leaf apex (Fig 9B), whereas DT14 is the removal of leaf tissue along the margin and to the primary vein (Fig 9A). DT15 is a deeper, trenched incision that is deeply incised and is parallel sided or expands towards the primary vein (Fig 9D), whereas DT198 also is a deep incision but narrows medially (Fig 9E). DT214 is a series of cuspate feeding traces with three or more, distinctively shaped, scalloped incisions (Fig 9F).

#### Skeletonization

**Fig 10:**
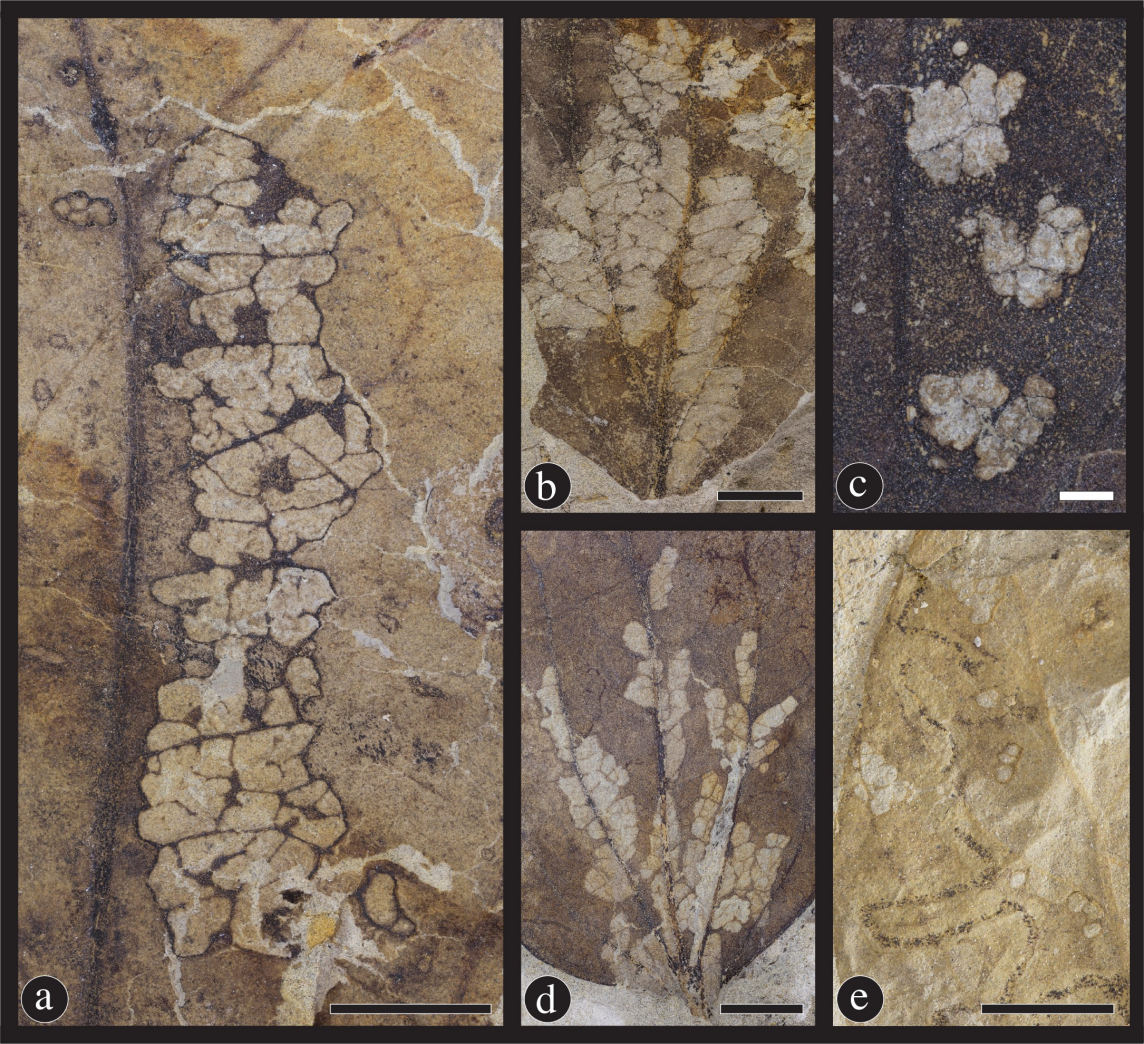
Skeletonization damage types on *Catula gettyi*. (A) DT22; Skeletonization which follows a primary vein (DMNH 41565); (B) DT19; Broad swaths of skeletonized tissue in a rectilinear shape (DMNH 41568); (C) DT24; Circular areas of skeletonization adjacent to the primary vein (DMNH 41578); (D) DT61; Elongate areas of skeletonization adjoining primary and secondary venation (DMNH 41591); and (E) DT16; Unadorned and common areas of tissue removal between veins (DMNH 41594). Black scale bars = 5 mm; white scale bars = 1 mm.

Skeletonization is similar to hole feeding in that there is consumption of the entire thickness of the leaf, but at least one order of venation remains intact, often creating a lace-like appearance. The five skeletonization damage types on *C*. *gettyi* are DT16, DT19, DT22, DT24, DT61 and DT333 (Fig 10). DT16 is the most frequently encountered skeletonization damage type and constitutes the nondescript removal of laminar tissue with veins remaining undamaged but lacking a distinct reaction rim of tissue produced by the plant host (Fig 10E). DT19 damage consists of elongate, rectilinear patches of skeletonized tissue with a length-to-width ratio of 2.5 or more (Fig 10B). DT22 are linear or curvilinear, elongate skeletonized areas parallel to and along the leaf margin that often have branching connections to each other (Fig 10A). DT61 is composed of an elongate swath of skeletonization that occurs on one side of a primary or secondary vein (Fig 10D); by contrast, DT24 are three or more circular, skeletonized areas adjacent to a primary or secondary vein (Fig 10C).

#### Surface feeding

**Fig 11:**
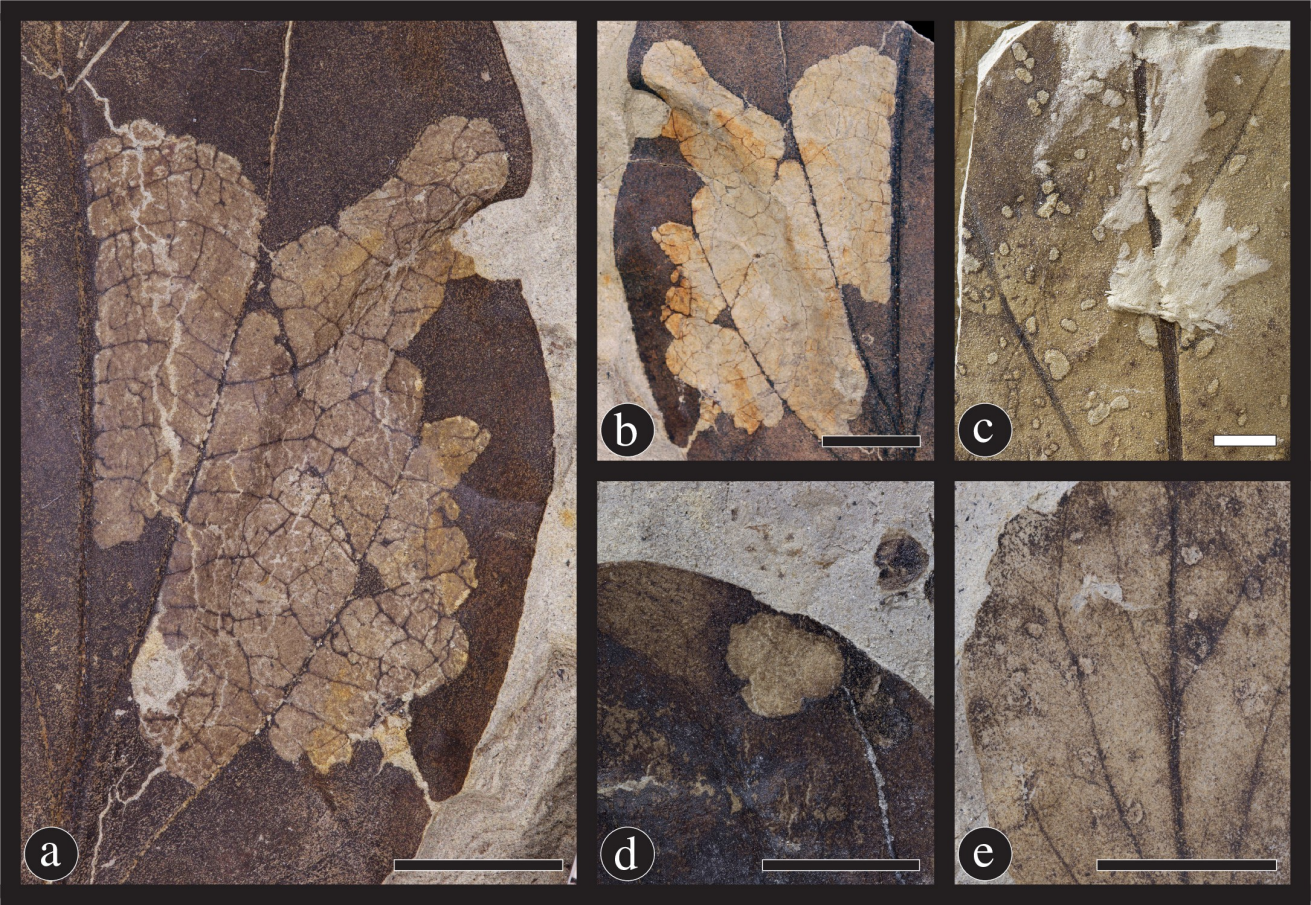
Surface feeding on *Catula gettyi* at the Lost Valley Locality. These damage types consist of three previously known damage types and one new damage type: (A,B) DT333 (DMNH 39725); This new damage type (DT) entails large areas of herbivory in which one surface of the leaf is removed and third-fourth order venation is left intact. Part A exhibits the rank of undamaged venation and does not have surface tissue removed. Counterpart B illustrates the tissue removal and the presence of intact third and fourth order veins. The other three surface feeding damage types are: (C) DT31; Removal of surface tissue with a distinct circular to ellipsoidal reactions rim (DMNH 41589); (D) DT30; Surface feeding with a polylobate reaction rim (DMNH 41566); and (E) DT29; Surface feeding with a weak reaction rim (DMNH 41593). Black scale bars = 5 mm; white scale bars = 1 mm.

Surface feeding is the consumption of one or more layers of surface tissues but not the entire blade thickness and occurs on either the abaxial or adaxial surface of the leaf lamina. The three examples of surface feeding damage types on *Catula gettyi* are DT29, DT30 and DT31, and one previously undescribed damage type of DT333 (Fig 11). DT29 is a commonly occurring, circular to polylobate area of surface-feeding damage that is recognizable by the absence of or minimal development of reaction tissue around the perimeter of the feeding zone (Fig 11E). In contrast, DT30 has a well-developed reaction rim with a polylobate margin bordering the surface abrasion patch (Fig 11D), whereas DT31 has a circular bordering margin and also a well-developed reaction rim (Fig 11C). The new surface feeding DT333 consists of polylobate surface abrasions nestled between primary and secondary veins, which leave primary, secondary, and third order venation intact (Fig 11A and 11B). This damage type is similar to some skeletonization damage types if the fossil leaf counterpart is not preserved; however, inspection of the undamaged side of the laminar tissue clearly reveals that surface feeding is confined to one surface of the leaf.

#### Piercing and sucking

**Fig 12:**
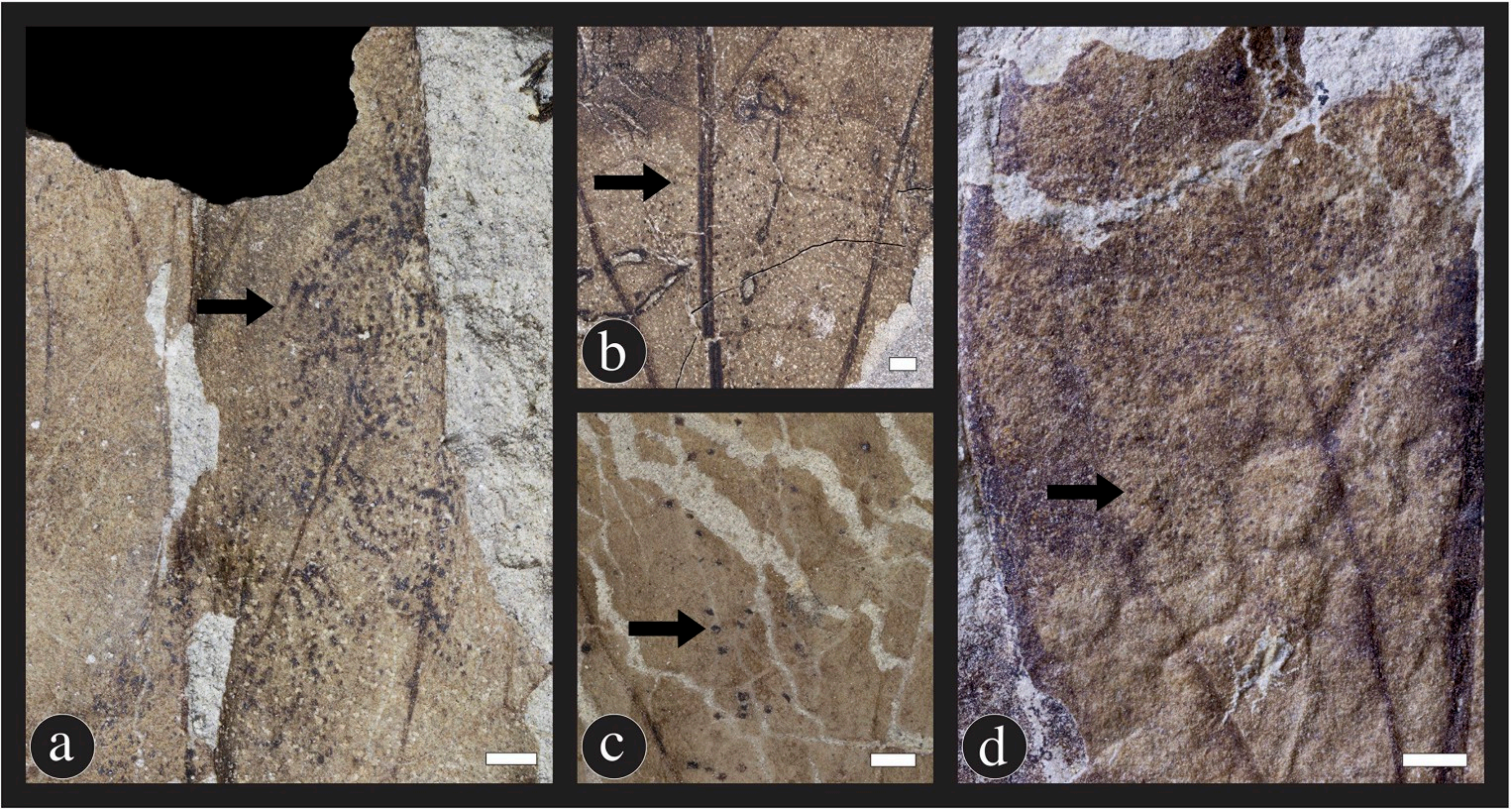
Insect damage caused by piercing-and-sucking insects. (A) DT219; This damage type (DT) consists of two mirrored lines of puncture marks, which are potentially made by mandibles (DMNH 39724). Additional evidence of piercing-and-sucking insects comes from circular, concave puncture marks DT46 (B) (DMNH 41590); and circular, convex puncture marks DT47 (C) (DMNH 41588). A second new piercing and sucking damage type (D) involves many puncture marks across large portions or the entirety of the leaf lamina (DT330) (DMNH 41569). Scale bars = 1 mm; arrows indicate a single puncture for clarity.

Piercing-and-sucking insects puncture and suck foliar tissues, such as epidermis, mesophyll, phloem and xylem. This fluid feeding is accomplished by use of mouthpart elements modified into elongate stylets, often encompassed by an external sheath. There are four piercing and sucking damage types on *C*. *gettyi*, including the two previously described damage types of DT46 and DT47, and the two recently described damage types of DT219 and DT330 (Fig 12). The most common piercing and sucking damage was DT46 (Fig 12B). This damage type consists of one to several concave punctures with a random distribution. DT47 includes many convex punctures that are irregularly distributed along and between secondary veins (Fig 12C). A newly described DT330 consists of a large number (> 50) of punctures covering a substantial portion of the lamina, frequently blanketing smaller areas in a dense sheet (Fig 12D) [60]. These punctures occur along veins of all ranks from primary to tertiary as well as vein inter-areas. These punctures differ from oil glands in that they are more irregular in size and shape and have a highly patchy distribution on the leaf surface.

The enigmatic DT219 is a distinct piercing-and-sucking pattern that consists of two parallel, mirrored lines of punctures indicating a directionality to movement as the putative insect moved across the lamina surface (Fig 12A). Although the identity of this insect herbivore remains unknown, it is possible that this feeding damage represents a sap feeder with incisiform mandibulate mouthparts. Because the punctures are paired and evenly spaced, they could be the result of paired mandibles puncturing the leaf surface, followed by ingestion of leaf exudate. Descriptions of this damage type were figured in two previous studies and redescribed herein. The first description of this unique damage type was from the Late Cretaceous of Israel [121], which was incorrectly diagnosed as a possible agromyzid leaf-mine damage. We find no evidence of mining for DT219. This type of damage was subsequently identified as surface feeding and described as paired mandibulate “chew marks” on *Araciphyllites tertiarius*, a monocot from the middle Eocene Messel Formation [122]. We agree with this latter description, but have reassigned this damage to DT219, within the piercing-and-sucking functional feeding group.

#### Oviposition

**Fig 13:**
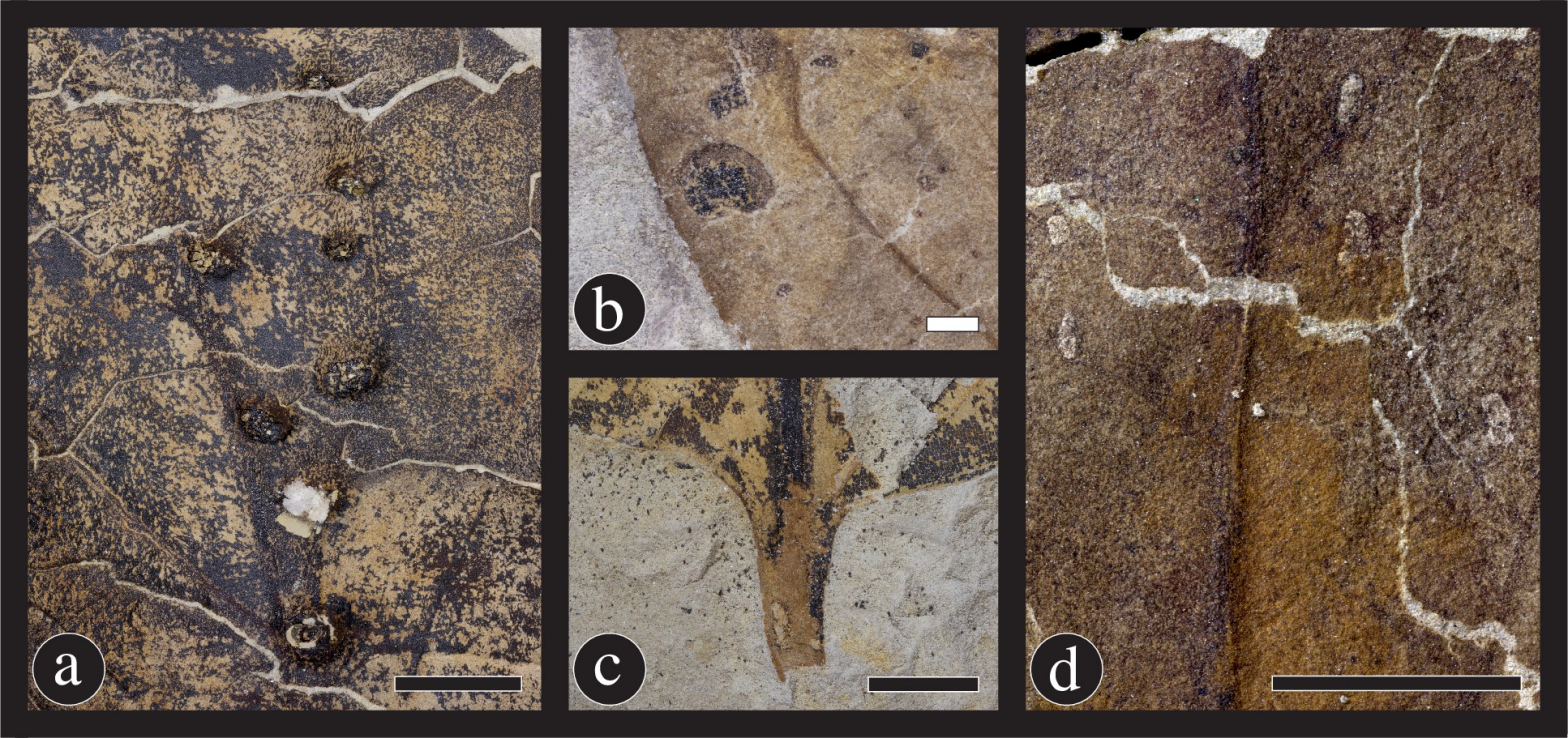
Galling and oviposition damage on *Catula gettyi*. (A) DT33 & DT34; Galls located on primary veins and secondary veins, respectively (DMNH 41577). (B) DT32; Galls located on the laminar surface, but avoiding primary and secondary venation (DMNH 41573). (C) DT85; Galls located on the petioles of leaves or petiolules of leaves (DMNH 41592). (D) DT101; Oviposition consists of multiple, scattered ovate-shaped scars produced by an insect bearing a robust ovipositor (DMNH 41570). Black scale bars = 5 mm; white scale bars = 1 mm.

Oviposition is the deposition of eggs into plant tissue, accomplished by a slicing or piercing insect ovipositor. Oviposition lesions are uncommon on *C*. *gettyi* leaves. There is one oviposition damage mode, DT101, with only three occurrences of all examined *C*. *gettyi* specimens (Fig 13D). All three occurrences are represented by one to four oval shaped lesions, replete with robust reaction rims.

#### Mining

**Fig 14:**
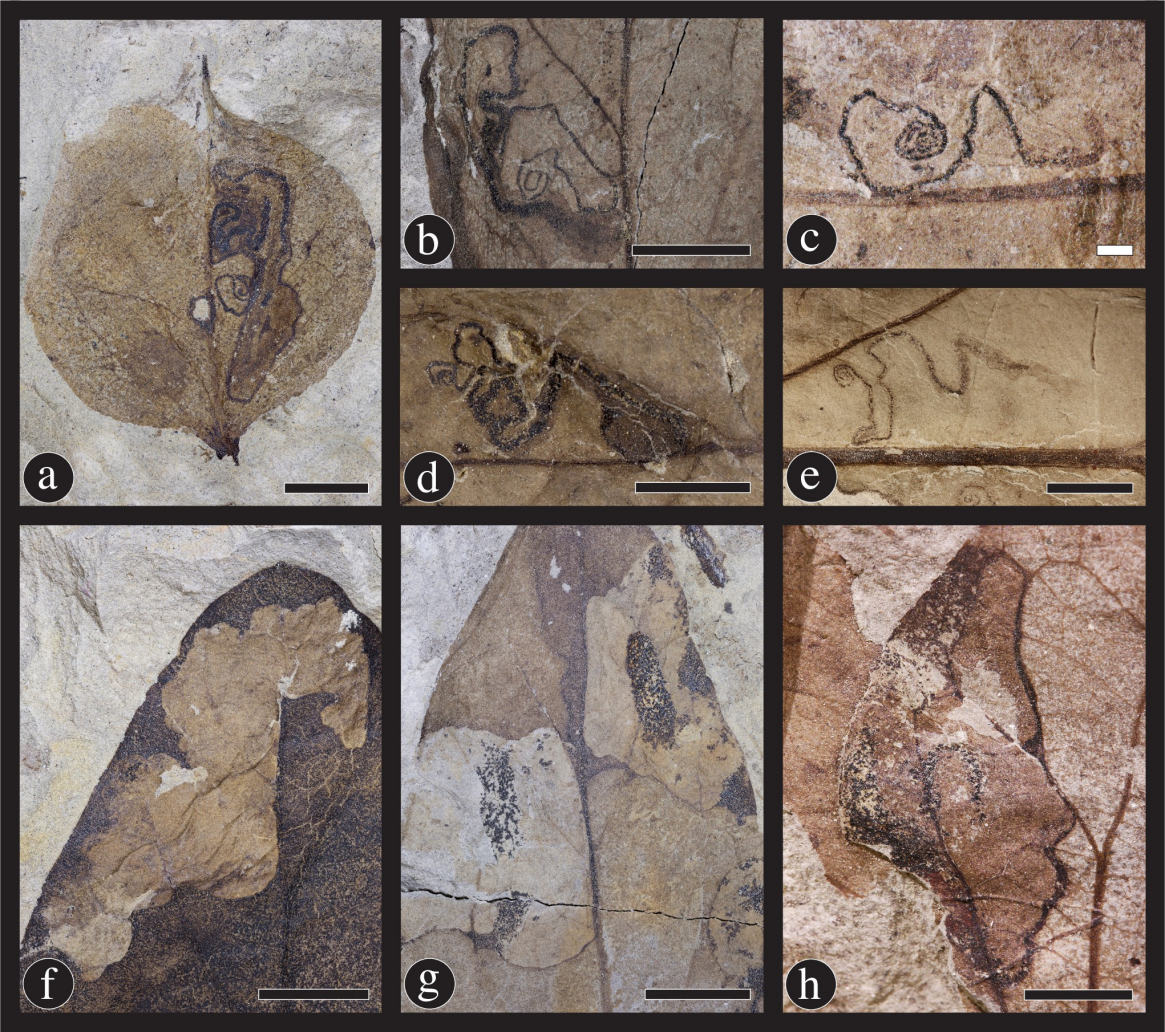
Mining insect damage at the Lost Valley Locality, Kaiparowits Formation, Utah. (A–E) DT45; Leaf mines attributed to the lepidopteran family Gracillariidae. These five mines illustrate the variation in overall shape, projection, and length, potentially due to the number of instars completed during the mining life history stage (A: DMNH 39732; B: DMNH 41582; C: DMNH 39735; D: DMNH 47124; E: DMNH 39733). The three types of blotch mines include: (F) DT36; A blotch mine lacking internal frass and a central chamber (DMNH 41572); (G) DT35; A blotch mine with frass present in circular central chamber (DMNH 41581); and (H) DT37; A blotch mine with an internal serpentine phase (DMNH 39734). Black scale bars = 5 mm; white scale bars = 1 mm.

**Fig 15:**
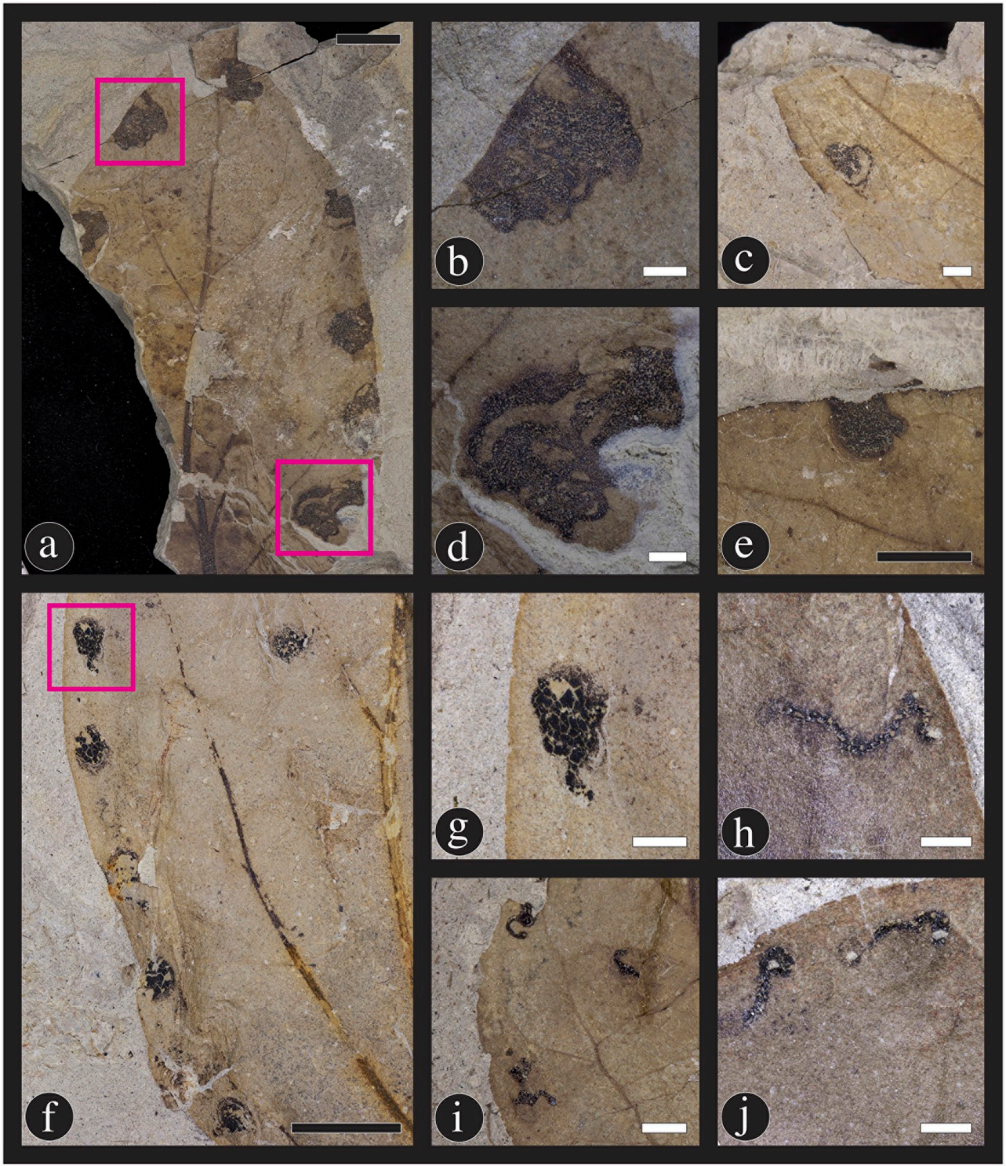
New leaf mine damage type on *Catula gettyi*. DT332 includes a range of leaf mine shapes, including (A) roughly circular mines with serpentine trajectories visible within the mine (DMNH 39726), close up of mines outlined (B, D); (E) leaf mines with a circular chamber and no evident serpentine trails (DMNH 41587); (C) a linear phase followed by a solid, circular chamber (DMNH 47126), (F) (DMNH 39737), close up of mine outlined (G,I) (DMNH 41597); and (H) (DMNH 41597), (J) (DMNH 41597) mines with linear trajectories. Differences in mine form are likely attributable to the number of instars completed by each individual. These mines are typically found along the margin of *C*. *gettyi* leaves. Black scale bars = 5 mm; white scale bars = 1 mm.

The most notable and distinctive insect damage type exhibited on *C*. *gettyi* are leaf mines, which are produced by several lepidopteran (moth) miners (Fig 14). There are three types of blotch mines, DT35, DT36 and DT37 (Fig 14F–14H), and the two serpentine mines of DT45 and DT332 on *C*. *gettyi* (Fig 14A–14E). The new leaf-mine DT332 is exceptionally abundant on this taxon, occurring on 112 leaf specimens, despite being previously unknown in the fossil record (Fig 15). The oviposition site for DT332 mines generally occurs along the leaf margin, and up to seven individual mines may occur on the same leaf specimen. The mines range from 1.4 mm to 7.1 mm at the broadest width of their mine trajectory. The earlier instars produce a minuscule, broadly serpentine shaped mine (Fig 15H–15J), while later instars produce a tightly sinuous, intestiniform pattern that often becomes blotch-shaped (Fig 15A–15G). Although the insect culprit is unknown, an analogous mine morphology currently appears on several plant hosts today, such as the leaf miners in the genera *Bucculatrix* Zeller 1839 (Lyonettidae) [123], which are similar in size, trajectory, and position compared to DT332 on *C*. *gettyi*. Based on overall similarities to modern mining moths, including mine size, non-overlapping mine trajectories and presence of solid frass, we posit that DT332 was created by a microlepidopteran leaf miner such as *Bucculatrix* or a related form [123].

The second, most common leaf-mine is DT45, with 122 specimens on *C*. *gettyi*, exhibiting one to four mines per leaf (Fig 14A–14E). This mine is attributed to the lepidopteran mining family Gracillariidae and was first described by Labandeira and colleagues [86,87] on specimens of the lauraceous *Marmarthia pearsonii* from the late Maastrichtian Hell Creek Formation (66 Ma) of the Williston Basin in North Dakota, USA. The DT45 mine on *C*. *gettyi* has a characteristic oviposition site and is initially thread-like and highly coiled, then is succeeded by repeated curvilinear phases, and ends in a sub-rectilinear to ovoidal terminal chamber. Frass is packed and is deployed continuously throughout the serpentine phases of the mine trajectory, with thick, modified bordering tissue constituting roughly 25% of the mine width on both sides of the frass trail. This mine generally is confined between primary and secondary veins, although this feature is variable on smaller leaves. DT45 varies substantially in size and length, which may be attributed to conspecific aborted mines and behavioral differences in larval instar activities.

Three blotch-mine damage types are found on *C*. *gettyi* leaves in the Lost Valley Locality. DT36 is the most frequently encountered of the blotch mine damage types (Fig 14F). The DT36 mine consists of variously shaped compartments that lacks a central chamber and sometimes contains spheroidal fecal pellets among the frass [123]. A similar mine is DT35, a blotch mine with a central chamber present, also commonly associated with spheroidal frass (Fig 14G). The third blotch mine, DT37, consists of a polylobate shaped blotch with an internal serpentine stage (Fig 14H).

#### Galling

Galls are envelopes of plant tissue that are induced and inhabited by insects, mites, nematodes, fungi, or bacteria. Insect galls generally consist of a hardened outer wall for protection and an inner layer of softer nutritive tissue connected to the host-plant organ by vascular tissue, all of which encapsulate an innermost chamber or chambers [76]. The four gall damage types on *C*. *gettyi* are located on the leaf lamina, consisting of nondescript DT32, DT33 and DT34 galls, and the petiole gall DT85 (Fig 13). DT32 consists of circular to ellipsoidal galls occurring on the leaf lamina and avoidance of major veins (Fig 13B). DT33 and DT34 represent galls similar in form to DT32, but instead occur on secondary veins and primary veins, respectively (Fig 13A). The distinctive DT85 is small, lenticular to ellipsoidal gall situated lengthwise along a midrib or petiole, with indistinct inner nutritive tissue and a thick, dark outer wall located on the petiole of *C*. *gettyi* (Fig 13C).

### Insect herbivory on late cretaceous laurels

**Fig 16:**
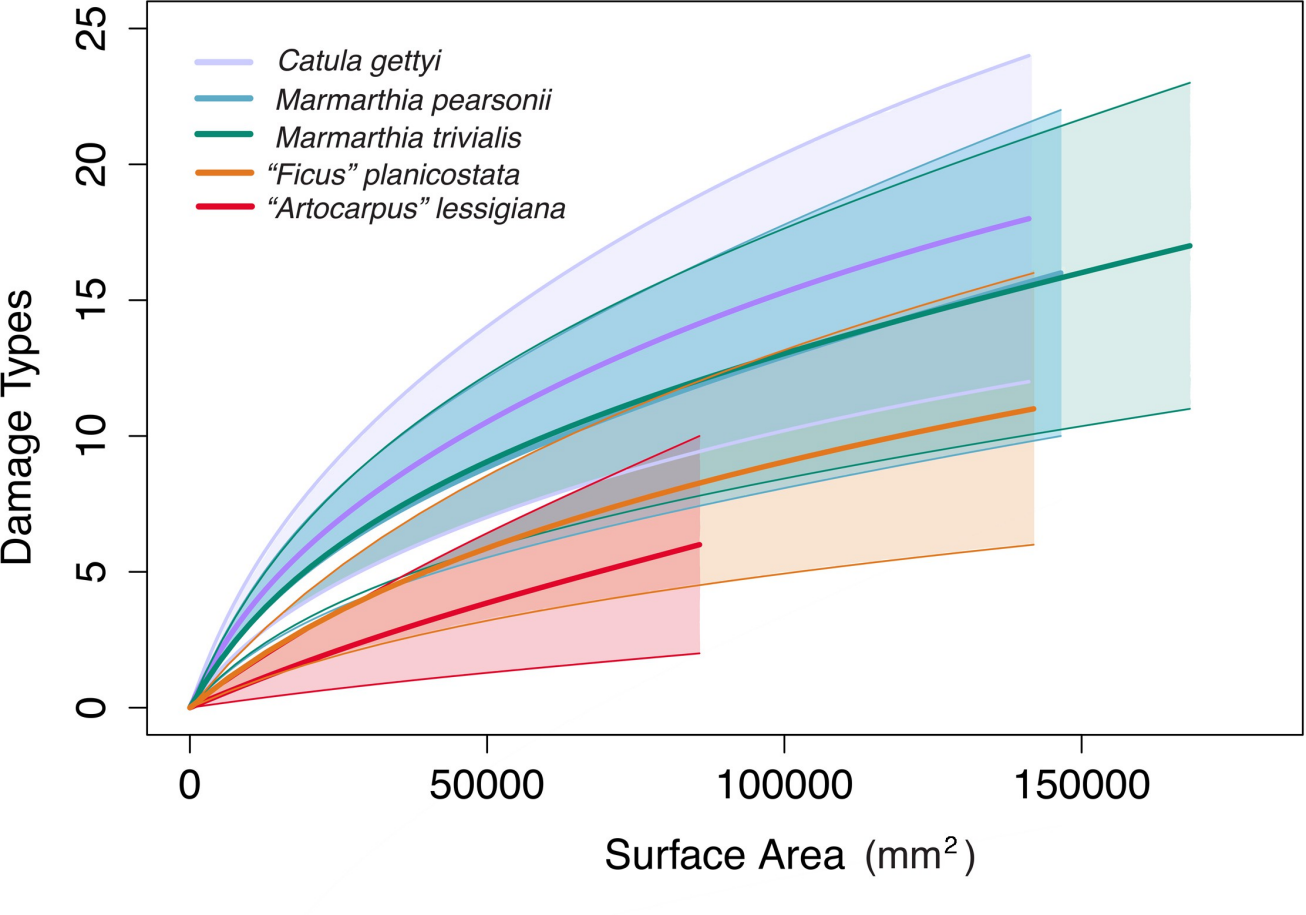
Rarefaction of damage types and total sampled surface area for *Catula gettyi* and the four Hell Creek taxa. The Hell Creek taxa are: *Marmarthia pearsonii*, *M*. *trivialis*, “*Artocarpus*” *lessigiana*, and “*Ficus*” *planicostata*.

The four Maastrichtian-aged laurel taxa (*Marmarthia pearsonii*, *M*. *trivialis*, *“Artocarpus” lessigiana*, and “*Ficus” planicostata*) and the Campanian-aged *Catula gettyi* had comparatively similar damage-type richness (Fig 16). The 84% confidence intervals of all five taxa overlapped in the rarefaction analysis for damage-type richness by total surface area. Intensities of insect herbivory, i.e. herbivory index, were also relatively similar among four of the five taxa (Table 2; S2 Fig). Herbivory indices ranged between 0.37% and 2.86%, with 95% confidence intervals overlapping for all taxa except *“A*.*” lessigiana*, which has a lower herbivory index than *C*. *gettyi* and *M*. *trivialis* (S2 Fig).

**Table 2:** Comparisons of herbivory between Late Cretaceous taxa in the family Lauraceae.

	*Catula gettyi* *	*Marmarthia trivialis*	*Marmarthia pearsonii*	*“Artocarpus” lessigiana*	*“Ficus” planicostata*
Number of specimens analyzed for surface area	156	67	167	32	23
Proportion of specimens with herbivory	40.76%	47.76%	20.40%	21.88%	60.87%
Total surface area (mm^2^)	142036.327	170748.468	147371.987	88484.531	148379.603
Herbivorized surface area (mm^2^)	2985.841	4886.361	1598.448	325.244	3921.650
Herbivory index	2.102%	2.862%	1.085%	0.368%	2.643%
Number of damage types	19	17	17	6	11

The taxa are: *Catula gettyi* (Kaiparowits Formation, 75.6 Ma), *Marmarthia pearsonii* (Hell Creek Formation, 66.5 Ma; Loc. 428), *M*. *trivialis* (Hell Creek Formation, 66.5 Ma; Loc. 900), “*Artocarpus*” *lessigiana* (Hell Creek Formation, 66.5 Ma; Loc. 428), and “*Ficus*” *planicostata* (Hell Creek Formation, 66.5 Ma; Loc. 428) [84].

*Randomly sampled 10% of total *Catula gettyi* specimens.

**Fig 17:**
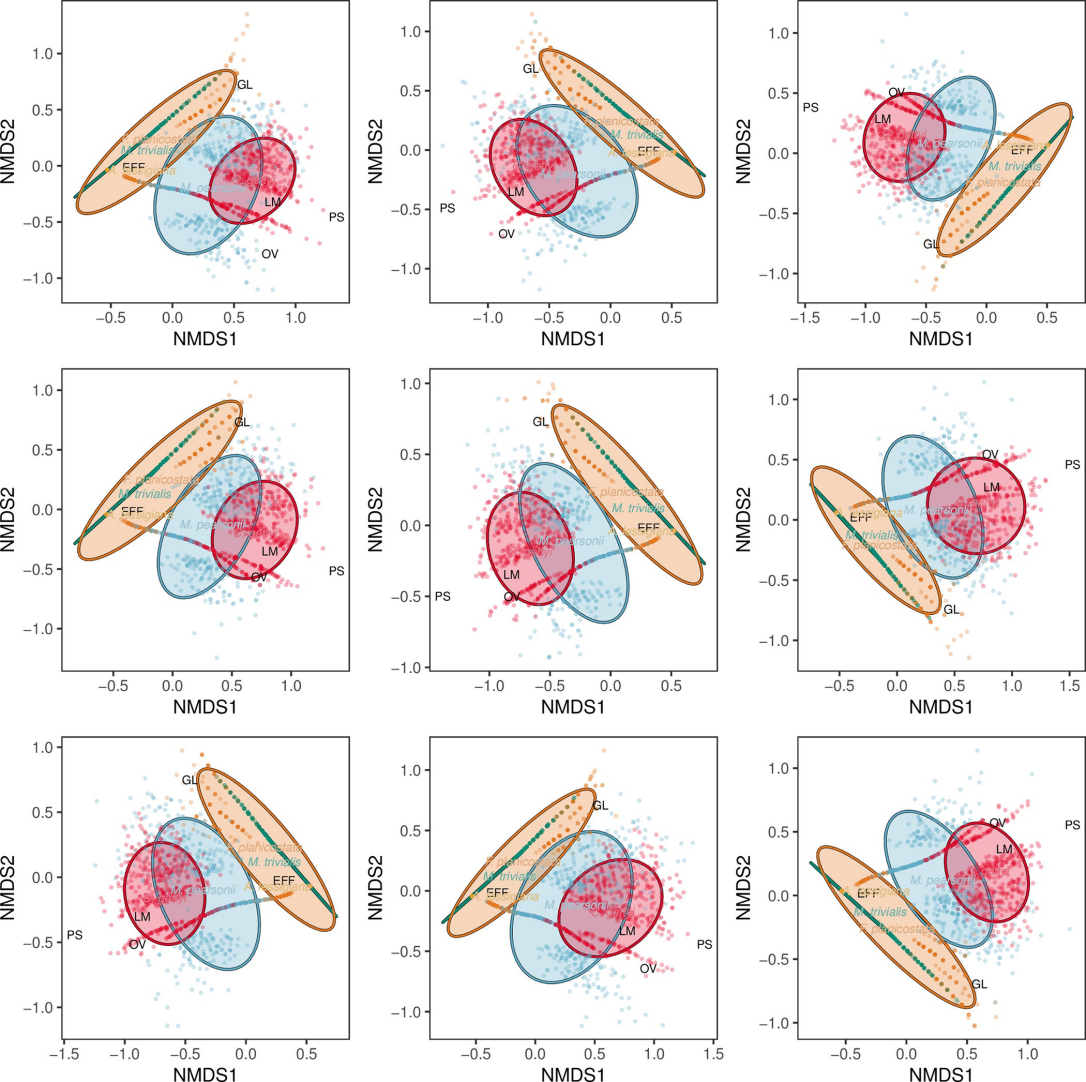
Non-metric multidimensional scaling (NMDS) ordination subsampled at 85,000 mm^2^. This ordination includes elipses, which denote 84% of the datapoints closest to the centroid, for *Catula gettyi* and the four Hell Creek taxa (*Marmarthia pearsonii*, *M*. *trivialis*, “*Artocarpus*” *lessigiana*, and “*Ficus*” *planicostata*).

**Fig 18:**
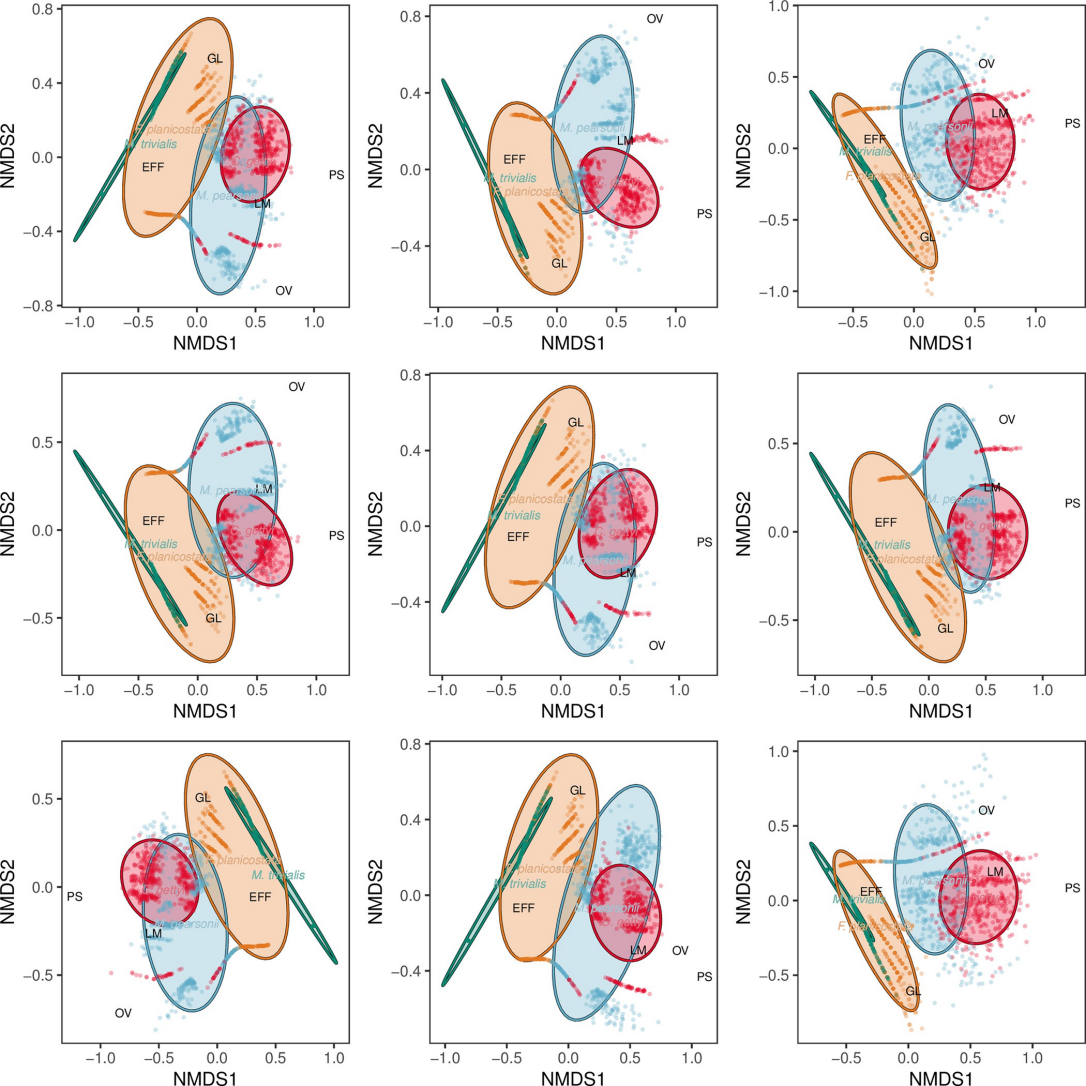
Non-metric multidimensional scaling (NMDS) ordination subsampled at 140,000 mm^2^. This ordination includes elipses, which denote 84% of the datapoints closest to the centroid, for *Catula gettyi* and three Hell Creek taxa (*Marmarthia pearsonii*, *M*. *trivialis*, and “*Ficus*” *planicostata*).

Finally, the spectrum of insect herbivory differed among the five laurel taxa (Figs 17 and 18). The nonmetric multidimensional scaling (NMDS) ordination plot that was subsampled to 85,000 mm^2^, which included all five taxa, illustrated a great deal of overlap between *C*. *gettyi* and *M*. *pearsonii* (Fig 17). Moreover, both *C*. *gettyi* and *M*. *pearsonii* were strongly associated with endophytic feeding groups of leaf mining and oviposition, as well as piercing and sucking. *Marmarthia trivialis*, “*F*.*” planicostata*, and *“A*.*” lessigiana* were tightly clustered in morphospace and associated with external functional feeding groups and gall makers. These patterns were more pronounced in the NMDS ordination plot that subsampled 140,000mm^2^, which clearly shows the overlap in morphospace between *C*. *gettyi* and *M*. *pearsonii*, and their shared association of leaf mining (Fig 18).

## Discussion

Six characters warrant the placement of *Catula*, *and C*. *gettyi*, within the Lauraceae and distinguish it from other fossil genera and species preserved in Early Cretaceous through Eocene strata: 1) simple leaves; 2) distichous and opposite or slightly subopposite leaf arrangement with axillary buds; 3) leaves with entire, or unserrated, margins; 4) pinnate primary venation; 5) simple brochidodromous secondary venation; and 6) a decurrent base with 1 or 2 pairs of acute basal secondary veins. Analysis of the leaf attachment, shape, and venation characteristics suggest that *C*. *gettyi* is allied with, but does not fall within, extant *Cinnamomum* as that genus exhibits better-organized higher order venation and arguably originates during the early Eocene [121]. Within *Cinnamomum*, *C*. *gettyi* is most similar to a venation syndrome that includes pinnate primary venation pattern with prominent basal secondary veins that have an acrodromous or brochidodromous course. In the distal portions of leaves of taxa that show this syndrome, additional well-defined brochidodromous secondary veins occur. Tertiary veins in this category are typically alternate percurrent to mixed opposite and alternate percurrent. These characters provide a more precise circumscription of the venation pattern that occurs in existing form genera and previous workers have considered simply as “triplinerved” [see 6]. To date, no floral or cuticular material assignable to *Catula gettyi* have been recovered, although future discoveries may expand its diagnosis. For the discussion accompanying the taxonomic description of *Catula gettyi*, please refer back to the Systematic section.

The damage intensity (herbivory index) and richness of insect damage on fossil leaves of *Catula gettyi* from the Kaiparowits Formation, combined with that of the latest Cretaceous Hell Creek Formation [86,87], provide a baseline to better understand Late Cretaceous herbivorous insect faunas and their associations with plants. Below, we discuss overall patterns of Kaiparowits insect herbivory, compare these results to other Late Cretaceous Lauraceae, and consider the impact of sampling effort and damage-type richness for insect herbivores in the Late Cretaceous fossil record.

### Kaiparowits formation insect richness

The plant–insect associations of the Kaiparowits Formation are moderately diverse on *Catula gettyi* leaves. We found evidence for eight functional feeding groups, 40 damage types, 863 occurrences of insect damage, an herbivory index of 2.102%, and 38.1% of *C*. *gettyi* leaves exhibiting insect herbivory. Interestingly, there are an elevated number of scale insect species on particular host species of extant Lauraceae [124–127] and the fossil record of scale insects ranges to the Permian [128–130], although no scale insects were found on *C*. *gettyi*. Specialized galls also occur on *Laurophyllum lanigeroides*, a laurel from the Eocene Messel Formation of central Germany [131], while galling damage on *C*. *gettyi* is relatively unspecialized and indistinct. By contrast, the frequency of leaf miners on modern Lauraceae are minimal [132] and occasionally reported in the fossil record [133–135], yet rich and abundant on *C*. *gettyi*. In general, the spectrum of insect damage on *Catula gettyi* is similar to that found on modern laurels (for a discussion on modern Lauraceae herbivory, see the Supporting Materials).

The distribution of host specificity was nearly evenly divided among damage types, wherein specialized (12 total), intermediate (15 total), and generalist (13 total) damage types each accounted for approximately one third of the total number of damage types. Nevertheless, the distribution of host specificity for individual damage type occurrences was bimodal (Fig 7), with greater generalist and specialist insect damage. Approximately half of all damage-type occurrences were assigned to the generalist category (51.9%), a small number were intermediate (8.8%), and specialist damage was also relatively high (39.3%). For qualitative categories, hole feeding (198 instances) and margin feeding (214 instances) had the most occurrences of generalist damage. Specialist damage was closely associated with endophytic feeding modes, most notable of which were the five leaf mining damage types constituting 311 occurrences, dominantly specialized in host specificity. The combination of high damage-type richness–including many distinct specialist damage types–suggests that *C*. *gettyi* likely hosted a rich community of insect herbivores.

In modern communities, endophytic insects most often make one, characteristic damage type on a single plant-host species [135–138]. This is because feeding behavior often is highly constrained and can sometimes be attributed to a taxonomic level of the subfamily, especially for endophytic feeders such as scale insects, leaf miners, and gallers [60,78,81]. Alternatively, ectophytic (chewing) insects are more likely to consume a wider range of host-plant species and often produce more numerous and diverse damage on leaves than their endophytic counterparts, which makes estimating ectophytic insect richness difficult in the fossil record [138]. Furthermore, a particular generalist damage type may be produced by multiple species of insects and, conversely, one species of insect may be capable of producing several damage types [138]. While we do not hypothesize an exact number of insect herbivores on *Catula gettyi*, we estimate that the specialized insect damage provides evidence for at least 12 specialist insect herbivores, based on the number of distinct associations from the total 40 damage types. For comparison, the number of arthropod herbivores on a single host-plant species in modern ecosystems varies greatly, with up to 205 phytophage species on leaves of certain taxa [ex. 139–142]. Our survey of *C*. *gettyi* captures many fewer herbivores. However, we were not able to measure fossil plant–insect associations with the same accuracy as modern plant–insect associations. Nevertheless, the observation of 40 damage types on a single fossil taxon is among the highest of any known fossil taxon [143,144], and the highest for a Cretaceous plant host.

### Antiherbivore resistance and herbivore specialization

Modern Lauraceae produce significant levels of secondary compounds and structural defenses. Many species of Lauraceae are noted for their elevated concentrations of essential oils that typically are employed in defenses against a wide range of insect herbivores today [145,146]. For example, lauraceous foliage is known to be rich in monoterpenes [147,148]; sesquiterpenes [148]; phenols of vanillic, chlorogenic, *p*-coumaric and ferulic acids [149]; as well as cyanoid diterpenes, extracts of cyandol, cyanoids and cinnceylanol [145]. These are known to have negative effects when fed to insects; the physiological outcomes of these extracts range from subtle antifeedant effects to toxins causing death [147]. Growth inhibition also is known for several lepidopteran (moth), coleopteran (beetle), and blattodean (termite) herbivores [145,150]. In addition to chemical defenses, Lauraceae possess considerable structural defenses. Features frequently found in Lauraceae indicating mechanical impediments to insect herbivory principally involve leaf toughness, such as thickened epidermis layers, cell-wall rigidity, thick cuticle, and robust fiber strands often associated with the vasculature [151]. As with the majority of modern Lauraceae, a combination of structural and chemical defenses likely was present in *C*. *gettyi* (also see Supporting Materials).

Although antiherbivore defenses in *Catula gettyi* can only be inferred, the morphology of the leaves suggests tough, long-lived leaves, similar to many extant Lauraceae species [152]. The leaves of *C*. *gettyi* have relatively thick petioles compared to their leaf area suggesting a high leaf mass per area quotient [153], however, quantitative measurements of leaf mass per area are needed to test this idea further. Furthermore, they show generally less physical damage, such as blade tearing and necrotic tissue, compared to other leaf morphotypes at the Lost Valley locality (SA Maccracken, pers. obs.). The antiherbivore defenses of long-lived leaves generally are constitutive (ever-present) and the metabolites are typically qualitative defenses, such as digestibility-reducers that are present at high levels [154], as compared to induced defenses [155]. These qualitative defenses are known to decrease the probability of insect herbivory from a wide range of both generalist and specialist insect herbivores [156,157] and lessening of fungal attacks [158]. Furthermore, long-lived leaves typically have lower photosynthetic rates and lower nitrogen content and greater structural tissues, which makes them less nutritious and palatable to insects [158]. The morphology of the leaves and the bimodal distribution of generalist and specialist damage types on *C*. *gettyi* are indicative of a plant species with constitutive defenses, such as the structural defenses that slow or prevent processing of leaf material by insect herbivores.

At present, secondary metabolites are poorly known in fossil leaves though efforts to detect and identify them are increasing [159–161]. Nevertheless, specialized insect damage types can be used, such as those made by leaf miners, to provide predictions about the role of secondary metabolites in *Catula gettyi*. A longstanding hypothesis is that elevated secondary compound defenses in plants often lead to taxon-specific coevolutionary relationships between the plant host and insect herbivore [162]. Specialist insects frequently have physical adaptations to their host plant’s secondary compounds, particularly involving tolerance, expulsion, or sequestration, although it is acknowledged that specialist herbivores also are negatively impacted by these toxins at high levels [163]. Moreover, insects instead use one or more particular toxic compounds as a cue to recognize potential plant hosts as edible or suitable as an oviposition site [164,165].

*Catula gettyi* hosts both abundant and diverse, specialized damage types, such as piercing and sucking, galling, and most notably mining. The elevated number of leaf-mine occurrences is exceptional in the fossil record compared to other plant host species [ex. 36,37,86,87,122,166,167]; 7.8% of *C*. *gettyi* specimens have the leaf mine DT45 and 7.2% have DT332, which are both attributed to lepidopteran miners. Lepidopteran leaf miners are well known for their abilities to disarm, digest, and/or tolerate plant secondary metabolites as an ever-present threat while living inside the leaf mesophyll [168]. The exceptionally high number of these two damage-type occurrences, the specialist nature of these leaf mines, and the lack of mines on other plant taxa from the same locality indicates that two leaf mining lepidopteran taxa were actively seeking out *C*. *gettyi* as an oviposition site for their larvae.

### Late cretaceous insect herbivory

Insect herbivory studies in the Mesozoic are lacking compared to those of the late Paleozoic and Cenozoic [49,169], with only a small number of Late Cretaceous floras having been analyzed for insect herbivory [170]. Among these studies, there are only a small number of descriptions for isolated Mesozoic damage types [ex. 38,59,78,81,166,171] and only two, fully described, insect damaged floras from the Late Cretaceous that predate the Kaiparowits Formation (Table 3). Of the two floras for which the damage has been fully described, the Soap Wash Formation of Utah (98.1 Ma) has a small sample size (152 specimens) [172], and the Ora Formation Flora of Israel (91 Ma) [37] does not use the damage type scheme. The lack of damage type designations, small sample sizes, and the approximation of specimen numbers and herbivory occurrences in the latter flora precludes a direct comparison of the Ora and Soap Wash formations to the Kaiparowits Formation regarding insect herbivory. Aside from these early Late Cretaceous deposits, only the Late Maastrichtian Hell Creek Formation in North America and the Lefipán Formation in South America, both of which are ca. 8–10 million years younger than the Kaiparowits Formation, provide a comparable Late Cretaceous dataset of insect herbivory.

**Table 3:** Late Cretaceous floras analyzed for herbivory.

Formation	State, Country	Age (Ma)	Number of Plant Taxa	Number of Specimens	Herbivorized Specimens	Number of DTs	References
Soap Wash	Utah, USA	98.4	18	152	64	19	Arens and Gleason [170]
Ora	Negev, Israel	~91	~50	~1500	N/A	~60*	Krassilov and Shuklina [35]
Kaiparowits	Utah, USA	~75.6	1	1564	606	40	*This study*
Hell Creek	North Dakota, USA	67–66	191	4149	657	32	Labandeira et al. [86,87]
Lefipán	Patagonia, Argentina	67–66	53	856	533	50	Donovan et al. [59]

*This study did not use damage type classification from Labandeira et al. [60], first employed in Labandeira et al. [86,87].

The Hell Creek and Lefipán Formation studies (Table 3) have yielded relatively high damage-type richness, between 32 and 60 damage types [59,86,87]. However, differences in habitat type, sampling intensity, sampling protocol, floral diversity, time averaging, deposit size, taphonomic variability, number of localities, and latitudinal position make comparisons among floras inequitable. Although comparisons between the Kaiparowits Formation insect herbivory and the insect herbivory in these other floras are inadvisable for these reasons, individual taxa from the Maastrichtian of North and South America collected from a single locality are analogous to the sampling of *C*. *gettyi* and therefore permit a more appropriate comparison. However, access to North American specimens was most feasible and geographically relevant. Given this context, we selected four taxa belonging to the family Lauraceae that had at least 20 specimens from a single locality in the Hell Creek Formation [84]: *Marmarthia pearsonii*, *M*. *trivialis*, “*Artocarpus*” *lessigiana*, and “*Ficus*” *planicostata* (Table 3) (Fig 3).

*Catula gettyi* has similar richness of damage types when compared to *Marmarthia pearsonii*, *M*. *trivialis*, *“Artocarpus” lessigiana*, and *“Ficus” planicostata* (Fig 16). Although *C*. *gettyi* has a high number of damage types across all 1,564 specimens, rarefaction analysis of damage-type richness by surface areas illustrates that the levels of insect damage are comparable to the Hell Creek laurels. Regarding the types of insect damage found on each taxon, *C*. *gettyi* and *M*. *pearsonii* are most tightly associated with miners (Figs 17 & 18). Notably, the specialist leaf mine DT45 only occurs on *C*. *gettyi* and *M*. *pearsonii* among the five lauraceous taxa, perhaps due to similar antiherbivore defenses. It is possible that this miner was well accommodated to the secondary metabolites of some Lauraceae species, as certain secondary metabolites shared between closely related plant taxa, known as semiochemicals or signal chemicals, deter lepidopteran oviposition [173]. However, this is speculative. DT45 also is present on morphotypes “LEF5” and “LEF9” from the Lefipán Formation, although their taxonomy currently is unresolved [59]. Future analyses comparing *C*. *gettyi* to other Late Cretaceous plants in Lauraceae and other angiosperm lineages should clarify the insect damage associations between these Late Cretaceous plant hosts. Indeed, we advocate for more taxonomic work in Late Cretaceous floras, coupled with ecological data for each plant host. This would particularly be useful for elucidating the forces driving patterns of Late Campanian regionalism and disparities in taxonomic richness observed in vertebrates [174–177] and invertebrates [178]. Latitudinally dispersed floras and associated indicators of insect richness from penecontemporaneous geologic units in the Western Interior are key to understanding how abiotic factors, such as sea level, climate, and tectonics influenced Late Cretaceous ecosystems.

## Conclusions

Herein we describe the new genus and species, *Catula gettyi* (Laurales: Lauraceae), from the Campanian Age Kaiparowits Formation of southern Utah, USA and catalog the insect damage on the taxon. With 1,564 studied museum voucher specimens, *C*. *gettyi* is among the best-sampled Mesozoic taxa in the fossil record for insect damage. Insect herbivory on *C*. *gettyi* is both rich and abundant, including eight functional feeding groups, 40 damage types, an herbivory index of 2.1%, and 38.1% of the specimens exhibiting at least one type of insect damage. There is a large damage component of generalist, ectophytic feeding as well as six specialist leaf miners. These results, in combination with the analysis of four Late Cretaceous lauraceous taxa from the Hell Creek Formation, show similar damage-type richness for these Late Cretaceous lauraceous plant hosts and possible specialization on lauraceous plant hosts.

Taken together, this first analysis showing the richness, abundance, and intensity of insect damage on a single taxon in the Kaiparowits Formation complements the high richness seen in vertebrates, invertebrates, and plants known from this geologic formation. Future work will assemble the insect–plant ecosystem and investigate how the base of the food web reflects diversity seen at higher trophic levels, including that of the diverse Kaiparowits Formation dinosaurs.

## Supporting information

S1 Fig Insect damage on Hell Creek taxa. (A–B) *Marmarthia pearsonii* leaf and closeup of leaf mine (DT45) (DMNH 7228, DMNH loc. 900), (C–D) *M*. *pearsonii* leaf and closeup of oviposition (DT54) (DMNH 7265, DMNH loc. 900), (E–F) *M*. *pearsonii* leaf and closeup of leaf mine (DT45) (DMNH 7199, DMNH loc. 900), (G–H) *M*. *trivialis* leaf and closeup of hole feeding (DT3) (DMNH 7495, DMNH loc. 428), (I–J) *M*. *trivialis* leaf and closeup of skeletonization (DT56) (DMNH 20165, DMNH loc. 428), (K–L) *“Ficus*” *planicostata* leaf and closeup of hole feeding (DT5) (DMNH 7567, DMNH loc. 428). White scale bars = 1.0 cm, black scale bars = 0.5 cm.
(TIF)

S2 Fig Herbivory indices for *Catula gettyi*, *Marmarthia pearsonii*, *M*. *trivialis*, “*Artocarpus*” *lessigiana*, and “*Ficus*” *planicostata*. Center line represents the herbivory index and the upper/lower boundaries represent the 95% confidence interval range.
(TIF)

S1 Table Comparable species to *Catula gettyi*. (DOCX)

S1 File Brief descriptions of herbivory and antifeedant properties of modern Lauraceae. (DOCX)
