# Correction: Insect herbivory on *Catula gettyi* gen. et sp. nov. (Lauraceae) from the Kaiparowits Formation (Late Cretaceous, Utah, USA)

**DOI:** 10.1371/journal.pone.0272757

**Published:** 2022-08-03

**Authors:** S. Augusta Maccracken, Ian M. Miller, Kirk R. Johnson, Joseph J. W. Sertich, Conrad C. Labandeira

The fourth author’s name is spelled incorrectly. The correct name is: Joseph J.W. Sertich. The correct citation is: Maccracken SA, Miller IM, Johnson KR, Sertich JJW, Labandeira CC (2022) Insect herbivory on *Catula gettyi* gen. et sp. nov. (Lauraceae) from the Kaiparowits Formation (Late Cretaceous, Utah, USA). PLOS ONE 17(1): e0261397. https://doi.org/10.1371/journal.pone.0261397.

## References

[1] Maccracken SA, Miller IM, Johnson KR, Sertich JM, Labandeira CC (2022) Insect herbivory on *Catula gettyi* gen. et sp. nov. (Lauraceae) from the Kaiparowits Formation (Late Cretaceous, Utah, USA). PLOS ONE 17(1): e0261397. doi: 10.1371/journal.pone.0261397 35061696 PMC8782542

