# Supplementary figures and images for: Insect herbivory on Catula gettyi gen. et sp. nov. (Lauraceae) from the Kaiparowits Formation (Late Cretaceous, Utah, USA)
Source: PLoS One. 2022 Jan 21;17(1):e0261397. doi: 10.1371/journal.pone.0261397 (PMC8782542; doi:10.1371/journal.pone.0261397)

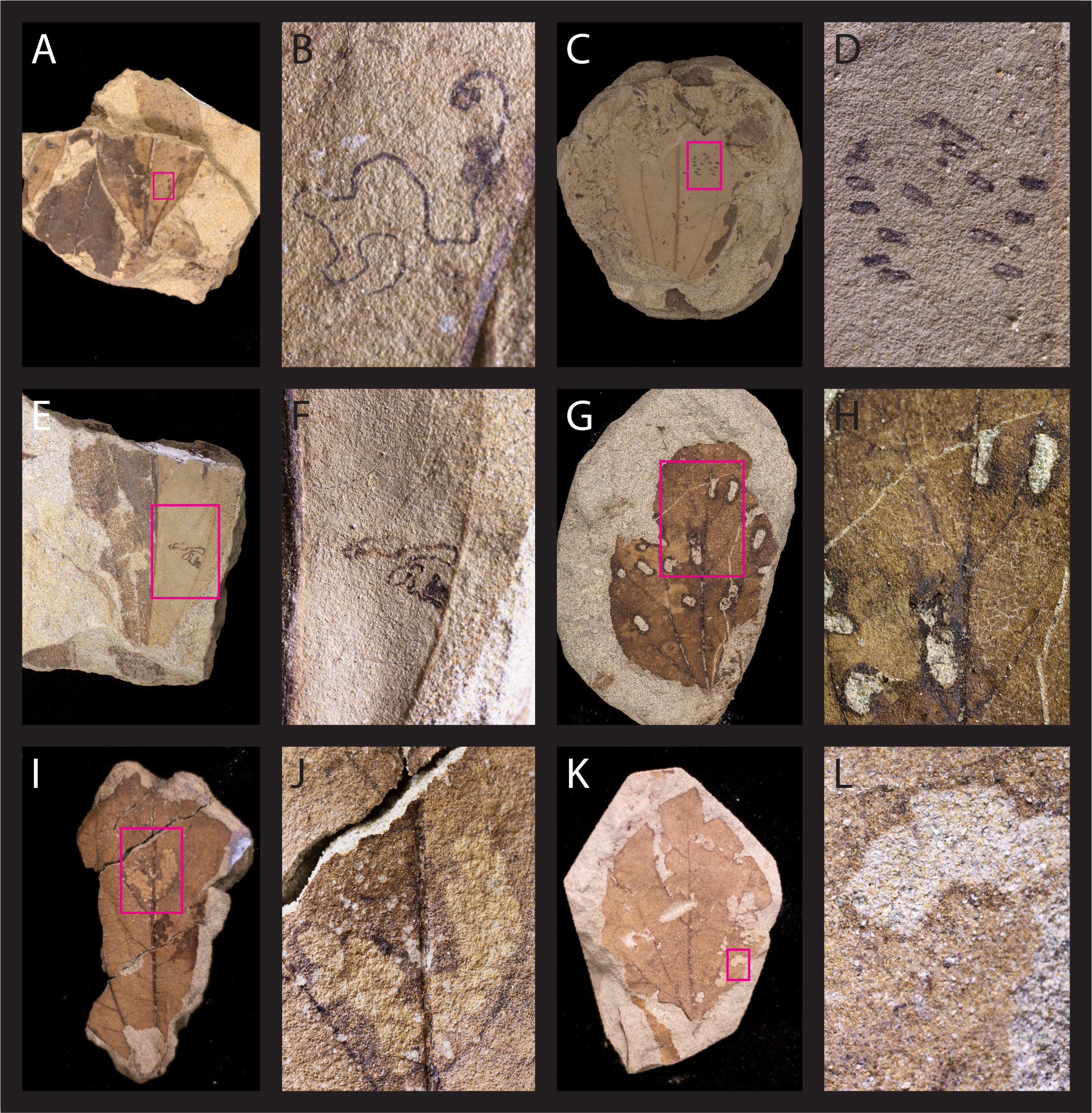

Supplement: S1 Fig — (A–B) Marmarthia pearsonii leaf and closeup of leaf mine (DT45) (DMNH 7228, DMNH loc. 900), (C–D) M. pearsonii leaf and closeup of oviposition (DT54) (DMNH 7265, DMNH loc. 900), (E–F) M. pearsonii leaf and closeup of leaf mine (DT45) (DMNH 7199, DMNH loc. 900), (G–H) M. trivialis leaf and closeup of hole feeding (DT3) (DMNH 7495, DMNH loc. 428), (I–J) M. trivialis leaf and closeup of skeletonization (DT56) (DMNH 20165, DMNH loc. 428), (K–L) “Ficus” planicostata leaf and closeup of hole feeding (DT5) (DMNH 7567, DMNH loc. 428). White scale bars = 1.0 cm, black scale bars = 0.5 cm. (TIF) [file pone.0261397.s001.tif]

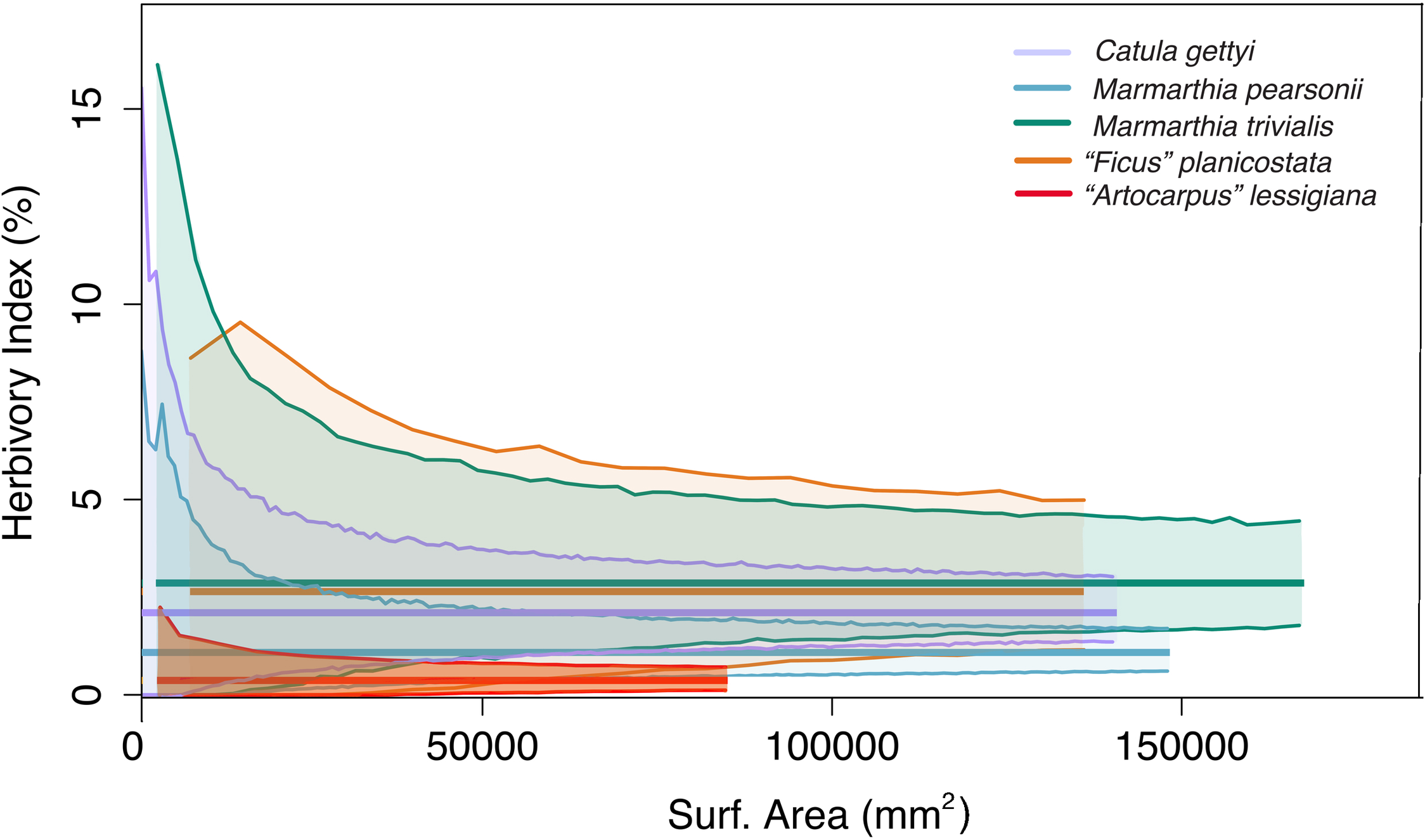

Supplement: S2 Fig — Center line represents the herbivory index and the upper/lower boundaries represent the 95% confidence interval range. (TIF) [file pone.0261397.s002.tif]
