# Supplementary material for: Insect herbivory on Catula gettyi gen. et sp. nov. (Lauraceae) from the Kaiparowits Formation (Late Cretaceous, Utah, USA)
Source: PLoS One. 2022 Jan 21;17(1):e0261397. doi: 10.1371/journal.pone.0261397 (PMC8782542; doi:10.1371/journal.pone.0261397)
Supplement: S1 Table — (DOCX) [file pone.0261397.s003.docx]

**S1 Table. Comparable species to *Catula gettyi.***

| **Species epithet** |
| --- |
| *Cinnamomum palaciosii* |
| *Cinnamomum hatschbahii* |
| *Cinnamomum litsaeaefolium* |
| *Cinnamomum halmaheirae* |
| *Cinnamomum pedatineruium* |
| *Cinnamomum australe* |
| *Cinnamomum glaziovii* |
| *Cinnamomum tomentulosum* |
| *Cinnamomum chana* |
| *Cinnamomum camphora* |
| *Cinnamomum montanum* |
| *Cinnamomum zapatae* |
| *Cinnamomum erythropus* |
| *Cinnamomum taubertianum* |
| *Cinnamomum pedunculatum syn. japonicum* |
