# Supplementary material for: Insect herbivory on Catula gettyi gen. et sp. nov. (Lauraceae) from the Kaiparowits Formation (Late Cretaceous, Utah, USA)
Source: PLoS One. 2022 Jan 21;17(1):e0261397. doi: 10.1371/journal.pone.0261397 (PMC8782542; doi:10.1371/journal.pone.0261397)
Supplement: S1 File — (DOCX) [file pone.0261397.s004.docx]

**Supplemental Materials for *Catula gettyi***

**Modern Insect Herbivory on Lauraceae**

Although all herbivore functional feeding groups provide a variety of damage types on extant members of the Lauraceae, there are some notable patterns of herbivory within the family. The DTs inflicted by hole, margin and surface feeders, as well as skeletonizers, are at levels of richness expected for a moderately diverse host-plant family such as Lauraceae. However, a considerable proliferation of associations exists between scale insect taxa and particular host species of Lauraceae. By contrast, the frequency of leaf miners on Lauraceae are minimal [1], while gallers are very diverse, particularly in the Neotropics [2]. Seed predation is overwhelmingly accomplished by small vertebrates rather than insects [3, 4]. Several ant–plant associations, such as between the sweetwood hosts *Ocotea* *dendrodaphne* Mez and *O*. *atirrensis* Mez & Donn.Sm and the ant symbiont *Myrmelachista* *flavocotea* Longino have been documented [5]. Another ant–plant association is between *Ocotea* *pedalifolia* Mez and the ant *Myrmelachista* sp., but involves a third mutualist member, the mealybugs *Dysmicoccus* *brevipes* (Cockerell) and *D*. *cryptus* (Hempel) that also inhabit the hollowed-out stems [6].

Hole Feeding. ― The polyphagous beet armyworm *Spodoptera* *exigua* Hubner (Lepidoptera: Noctuidae) is a prominent hole feeder on *Lindera* *benzoin* L. (spicebush) in the Southeastern United States. This generalist herbivore, however, lessens its levels of hole feeding under conditions of induced response in which its plant host responds to herbivore attack by producing feeding-deterrent substances (antifeedants) and structural defenses in foliar tissues [7, 8].

Margin Feeding. ― The specialist herbivore, *Epimecis* *hortaria* F. (Lepidoptera: Geometridae) is a major margin feeder on *Lindera* *benzoin* L. and *Sassafras* *albidum* (Nuttall) Nees (white sassafras). This relationship is particularly striking, as elevated levels of photoprotective phenolic compounds in sun leaves where higher temperatures increase photosynthesis [7], result in decreased nitrogen levels. Larvae of *E. hortaria* that are fed sun leaves respond by increasing significantly their level of herbivory [8]. By contrast, shade leaves on the same plant display decreased levels of *E*. *hortaria* margin feeding. although this pattern is not true for all herbivores [9]. The Promethea Silkmoth, *Callosamia* *promethea* (Lepidoptera: Saturniidae), is a large caterpillar that feeds on *Sassafras* *albidum* as one of its primary hosts, also preferring sun leaves over shade leaves [10].

Skeletonization. ― Although recently introduced from Japan, the highly polyphagous Japanese beetle *Popillia* *japonica* (Coleoptera: Scarabaeidae) is probably the most prolific skeletonizer of white sassafras, *Sassafras* *albidum*, a native to Eastern North America [11]. Other than the Japanese beetle, there are few native skeletonizing insects on Lauraceae.

Surface Feeding. ― Several species of the specialist skipper genus *Venada* (Lepidoptera: Hesperiidae) apparently are obligate surface feeders of several species of the aromatic, lauraceous *Ocotea* (sweetwood) [12]. The feeding damage of *Venada* produces light-colored, polygonal patches of removed epidermal leaf tissue. The most conspicuous obligate surface feeder on Lauraceae is the Spicebush Swallowtail, *Papilio* *troilus* L. (Lepidoptera: Papilionidae), whose larvae feed on members of Lauraceae in Eastern North America such as *Lindera* *benzoin*, *Sassafras* *albidum*, its two primary hosts, and *Cinnamomum* *camphora* L. J. Presl. (camphor), *Persea* *borbonia* L. (Spreng.) (redbay), and occasionally members of more distantly related Magnoliaceae [13].

Piercing and Sucking. ― The numerous relationships between piercing-and-sucking scale insects and planthoppers on modern Lauraceae hosts indicate that piercing and sucking harbors the single most taxonomically diverse functional feeding group for Lauraceae. Almost all of these common hemipteran associations are polyphagous and target vascular phloem tissue, such as the black scale, *Saissetia* *oleae* (Bernard) (Coccidae), on California bay *Umbellaria* *californica* (Hook. & Arn.) Nutt. in California. The similarly polyphagous cottony cushiony scale, *Iceyra* *purchasi* Maskell (Monophlebidae), is found on bay laurel *Laurus* *nobilis* L., as does the planthopper, *Metcalfa* *pruinosa* (Say) (Flatidae), also occurring on *L.* *nobilis*. The palm fiorina scale, *Fiorinia* *fioriniae* Targioni-Tozzetti (Diaspididae), is a worldwide pest on species of *Persea*, particularly avocado, *P*. *americana* Mill., in California and Florida, although palms are the preferred host. Piercing-and-sucking insects produce damage on their host plants characterized as cratered punctures and circular, elliptical or ovoidal impressions on surface tissues [14].

Oviposition. ― Many Lepidoptera, such as papilionid butterflies, oviposit on the leaf surfaces of a variety of lauraceous hosts that often are food plants for their larvae [15, 16]. However, these oviposition events do not leave detectible damage on epidermal tissue and would not enter the fossil record. There are few instances of documented oviposition that produce recognizable scar tissue on extant Lauraceae. One example is the damselfly *Palaemnema* *desiderata* Selys (Odonata: Platystictidae) ovipositing on the twigs, branches and leaf petioles of the lauraceous *Licaria* sp. in Panama [17].

Mining. ― Lauraceae hosts are poorly represented among leaf-mining insects. The most prominent leaf-miner family on Lauraceae [18, 19] are the gracillariid genera *Acrocercops*, *Caloptila*, *Gracillaria*, *Lithocolletis* and *Phyllocnistis* that occur on *Cinnamomum*, *Laurus*, *Lindera*, *Litsea*, *Persea*, *Sassafras* and *Umbellaria* [20-26]. Documented occurrences of the yellow poplar weevil, *Odontopus* *calceatus*, also termed the Yellow Poplar Weevil and Sassafras Weevil (Coleoptera: Curculionidae), mines *Liriodendron* *tulipifera* L. (tuliptree), its primary host, but also mines less commonly *Sassafras* *albidum* (Nuttall) Nees and introduced *Laurus* *nobilis* L. in Florida [27]. The apple leaf miner, *Lyonetia* *clerkella* L. (Lepidoptera: Lyonettidae), occasionally occurs on *Laurus* *nobilis*, although its primary host are Rosaceae and birch across Eurasia and Northern Africa [28]. Although no leafmining flies (Diptera: Agromyzidae) are leaf miners of Lauraceae, at least 13 genera of Lauraceae host at least four *Phytobia* species of cambium miners in stems and trunks [1].

Galling. ― The most notable, described gall association is the avocado psyllid *Trioza* *anceps* Tuthill (Hemiptera: Psyllidae) on avocado, *Persea* *americana* L., in the southeastern United States. The incidence of this leaf-curl gall is dependent on the specific chemical profile of its host plant, preferring the variety *drymifolia* within populations of *P*. *americana* [29]. Another *Trioza* induced gall occurs on *Nectandra* *salicina* in Costa Rica, resulting in malformation of a pedunculated fruit that becomes enlarged and is sessilely positioned at the base of a twig [30]. One of several Cecidomyiidae (gall midge) galls on Lauraceae is *Pseudasphondylia* *neolitseae* (Diptera: Cecidomyiidae), a conical foliar gall on *Neolitsea* *seriacea* (Blume) Koidz. (Japanese Silver Tree) in southern Kyushu, Japan [31]. Nevertheless, the greatest diversity of galls on Lauraceae are present in the Neotropics [2]. Hosts include *Cinnamomum* (cinnamon), *Cryptocarya* (mountain laurel), *Nectandra* (sweetwood), *Ocotea* and probably unaffiliated genera [2, 32, 33].

Seed Predation. ― Seeds of Lauraceae are overwhelmingly large and typically are dispersed and occasionally predated by birds and small mammals [4, 34]. Insect predation on seeds of Lauraceae is rare and typically affect species with smaller seeds. One example is loblolly sweetwood *Ocotea* *leucoxylon* (Sw.) Laness. occurring on landslides within forests in Puerto Rico, that are predated by undisclosed insects [3]. The large, robust fruits of many species of *Persea* (bay, avocado) that are distributed in the Neotropics have a dispersal syndrome favoring intact consumption and dispersal of the large seeds by mammal megaherbivores that became extinct during the end of the Pleistocene Epoch [35]. The extinction of browsing and seed-dispersing megaherbivores such as litopterns, ground sloths, gompotheres and New World horses rendered many modern species of *Persea* as “neotropical anachronisms” that presently lack the facility for seed dispersal [36].

5.2.10. Pathogens. ― Laurel wilt is a vascular disease that invades the xylem of many species of Lauraceae. The disease caused by *Raffaelea* *lauricola* T.C. Harr (Ascomycetes: Ophiostomataceae) and is vectored by the ambrosia beetle *Xyleborus* *glabratus* Eichoff (Coleoptera: Curculionidae) and is especially harmful to *Persea* *borbonia* of the Southeastern United States [37].

Tritrophic Interactions. ― When generalist (polyphagous) lepidopteran larvae of Arctiidae and Megalopytidae that consumed secondary compounds of *Nectandra* *hypoleuca* and *N*. *latifolia*, respectively, were in turn consumed by the adult ant *Paraponera* *clavata*, there was a minimal rejection rate of the larval prey items [38]. However, when specialist (monophagous) lepidopteran larvae of Lasiocampidae, Nymphalidae and Saturniidae consumed *N*. *hypoleuca*, *Ocotea* *meziana* and *Ocotea* sp., respectively, there was an elevated rejection rate of the larval prey items. This study indicates that specialist lepidopteran larvae feeding on certain species of Lauraceae can sequester and concentrate secondary compounds to the detriment of predators such as an ant.

**Antiherbivore Resistance in Modern Lauraceae.**

Modern Lauraceae contain significant levels of secondary compounds. For example, *Persea* *americana* Mill. foliage is especially rich in monoterpenes, sesquiterpenes and similar compounds [39] in the Southeastern United States. In Eastern North America *Lindera* *benzoin* has low levels of herbivory particularly in sun rather than shade environments due to production of the phenols of vanillic, chlorogenic, *p*-coumaric and ferulic acids [40]. Monoterpenes occur at modest levels at all developmental stages of California bay, *Umbullaria* *californica* (Hook. & Arn.) Nutt, where it apparently is a deterrent to folivorous insects and blacktail deer [41], but prized as adding flavor and aroma to cooked foods. However, at the Miyazaki Experimental Forest in Japan, foliar extracts of 16 lauraceous species of *Actinodaphne*, *Cinnamomum*, *Laurus*, *Lindera*, *Litsea* and *Machilus* proved to have negative consequences when fed to insects [42, 43]. The outcomes of these extracts ranged from subtle antifeedent effects to toxins causing death [42, 43].

An interesting occurrence in the Canary Islands of Spain features a laurel forest that is highly defended chemically, where *Appolonia* *barbusana*, *Laurus* *azorica*, *Ocotea* *foetens* and *Persea* *indica* exhibit elevated levels of cyanoid diterpenes that have varying levels of insecticidal properties [42, 43]. Cyanoid dipertene plant extracts of cyandol, cyanoids and cinnceylanol from the four lauraceous genera exhibited modest antifeedant properties to strong growth inhibition when fed to larvae of the lepidopteran tobacco cutworm *Spodoptera* *litura* F. (Noctuidae), cotton bollworm *Heliothis* *armigera* Hübner (Noctuidae) and tussock moth *Calliteara* *fortunata* Rogenhofer (Erebidae), as well as the Japanese termite *Reticulitermes* *speratus* (Kolbe) (Isoptera: Rhinotermitidae) [42, 43]. The moderate to strong insecticidal properties of extracts from these four lauraceous species suggest a collective response to insect herbivory in a geographically constrained, insular environment. Interestingly, in a DNA–bar-coding study of a rainforest dominated by Dipterocarpaceae and from the much larger island of Borneo, species of the leaf beetle *Anadimonia* that were specialized on two species of Lauraceae displayed low levels of feeding, indicating high levels of chemically-defended lauraceous foliage [44].

In addition to chemical defenses, Lauraceae possess considerable structural defenses. Features frequently found in Lauraceae indicate mechanical impediments to insect herbivory that principally involve leaf toughness [45]. Elements contributing to leaf toughness are thickened epidermis layers, cell-wall rigidity in hypodermis layers, and the presence of robust, girdling fiber strands [45]. Recently, the report of cork warts on the leaves of six species of *Mezilaurus* indicates a novel type structural defense in Lauraceae [46]. Cork warts are accumulations of suberized, thickened cells surrounding smaller, radially arranged epidermal cells [46], and provide an additional level of antiherbivore structural defense.

1. Spencer KA. Host specialization in the world Agromyzidae (Diptera): Springer Science & Business Media; 2012. 443 p.

2. Maia VC, Carvalho-Fernandes SP, Rodrigues AR, Ascendino S. Galls in the Brazilian Coastal Vegetation. Neotropical Insect Galls: Springer; 2014. p. 295–361.

3. Myster RW. Seed predation, disease and germination on landslides in Neotropical lower montane wet forest. Journal of Vegetation Science. 1997;8(1):55–64.

4. Martins AM, Abilio FM, de Oliveira PG, Feltrin RP, de Lima FSA, Antonelli PO, et al. Pondberry (*Lindera melissifolia*, Lauraceae) seed and seedling dispersers and predators. Global Ecology and Conservation. 2015;4:358–68.

5. McNett K, Longino J, Barriga P, Vargas O, Phillips K, Sagers CL. Stable isotope investigation of a cryptic ant-plant association: *Myrmelachista flavocotea* (Hymenoptera, Formicidae) and *Ocotea* spp.(Lauraceae). Insectes Sociaux. 2010;57(1):67–72.

6. Stout J. An association of an ant, a mealy bug, and an understory tree from a Costa Rican rain forest. Biotropica. 1979;11(4):309–11.

7. Niesenbaum RA, Kluger EC. When studying the effects of light on herbivory, should one consider temperature? The case of *Epimecis hortaria* F.(Lepidoptera: Geometridae) feeding on *Lindera benzoin* L.(Lauraceae). Environmental Entomology. 2006;35(3):600–6.

8. Mooney E, Niesenbaum R. Population‐specific responses to light influence herbivory in the understory shrub *Lindera benzoin*. Ecology. 2012;93(12):2683–92.

9. Niesenbaum RA. The effects of light environment on herbivory and growth in the dioecious shrub *Lindera* *benzoin* (Lauraceae). American Midland Naturalist. 1992:270–5.

10. Osier T, Jennings S. Variability in host‐plant quality for the larvae of a polyphagous insect folivore in midseason: The impact of light on three deciduous sapling species. Entomologia Experimentalis et Applicata. 2007;123(2):159–66.

11. Potter DA, Held DW. Biology and management of the Japanese beetle. Annual Review of Entomology. 2002;47(1):175–205.

12. Burns JM, Janzen DH, Hallwachs W, Hajibabaei M. DNA barcodes reveal yet another new species of *Venada* (Lepidoptera: Hesperiidae) in northwestern Costa Rica. Proceedings of the Entomological Society of Washington. 2013;115(1):37–48.

13. Nitao JK, Ayres MP, Lederhouse RC, Scriber JM. Larval adaptation to lauraceous hosts: Geographic divergence in the spicebush swallowtail butterfly. Ecology. 1991;72(4):1428–35.

14. Johnson WT, Lyon HH. Insects that feed on trees and shrubs. Comstock Pub. Associates, Ithaca NY. 1991.

15. Carter M, Feeny P. Host-plant chemistry influences oviposition choice of the spicebush swallowtail butterfly. Journal of Chemical Ecology. 1999;25(9):1999–2009.

16. Frankfater CR, Scriber JM. Florida red bay (*Persea borbonia*) leaf extracts deter oviposition of a sympatric generalist herbivore, *Papilio glaucus* (Lepidoptera: Papilionidae). Chemoecology. 1999;9(3):127–32.

17. Soriano EG, Gutierrez RN, Garza MV. Reproductive behavior of *Palaemnema desiderata* Selys (Odonata: Platystictidae). Advances in Odonatology. 1982;1(1):55–62.

18. Labandeira CC, Johnson KR, Wilf P. Impact of the terminal Cretaceous event on plant–insect associations. Proceedings of the National Academy of Sciences of the United States of America. 2002;99(4):2061–6.

19. Labandeira CC, Johnson KR, Lang P. Preliminary assessment of insect herbivory across the Cretaceous-Tertiary boundary: major extinction and minimum rebound. Geological Society of America Special Paper. 2002;361:297–327.

20. Fletcher TB. Life-histories of Indian Insects: Microlepidoptera. 1920. Privately published, 85 p.

21. Fletcher TB. Life-histories of Indian Microlepidoptera (Second Series) Cosmopterygidae to Neopseustidae1933. Privately published, 217 p.

22. Needham JG, Frost SW, Tothill BH. Leaf-mining insects. Baltimore, Maryland: The Williams & Wilkins Co.; 1928. 351 p.

23. Hering E. Bestimmungstabellen der Blattminen von Europa, band I. W Junk, s’ Gravenhage. 1957:306–9.

24. Kumata T. A taxonomic revision of the *Gracillaria* Group occurring in Japan (Lepidoptera: Incurvarioidea). Insecta Matsumurana. 1982;26:1–186.

25. Kumata T, Kuroko H, Ermolaev V. Japanese species of the *Acrocercops* Group (Lepidoptera: Gracillariidae) Part 2. Insecta Matsumurana. 1988;40:1–133.

26. Yuan D, Robinson GS. *Caloptilia* leaf-miner moths (Gracillariidae) of south-east Asia. Bulletin of the British Museum (Natural History), Entomology Series. 1993;62(1):37.

27. Buss E. Leafminers on ornamental plants. Revised edition. Gainesville, Florida: Florida Cooperative Extension Service, Institute of Food and Agricultural Sciences, 2006.

28. Berg W. Zur Kenntnis der Obstbaumminiermotte *Lyonetia clerkella* L. unter besonderer Berücksichtigung des Massenwechsels während der Jahre 1951 bis 1953. Zeitschrift für Angewandte Entomologie. 1960;45(3):268–303.

29. Torres-Gurrola G, Delgado-Lamas G, Espinosa-García FJ. The foliar chemical profile of criollo avocado, *Persea americana* var. *drymifolia* (Lauraceae), and its relationship with the incidence of a gall-forming insect, *Trioza anceps* (Triozidae). Biochemical Systematics and Ecology. 2011;39(2):102–11.

30. Blackmer J, Hanson P. Abundance and life history of two gall-inducing homopterans on *Nectandra salicina* (Lauraceae) in Monteverde, Costa Rica. Revista de Biología Tropical. 1997:1131–7.

31. Yukawa J, Akimoto K. Influence of synchronization between adult emergence and host plant phenology on the population density of *Pseudasphondylia neolitseae* (Diptera: Cecidomyiidae) inducing leaf galls on *Neolitsea sericea* (Lauraceae). Population Ecology. 2006;48(1):13–21.

32. Julião GR, Almada ED, Fernandes GW. Galling insects in the Pantanal wetland and Amazonian rainforest. Neotropical Insect Galls: Springer; 2014. p. 377–403.

33. Medianero E, Barrios H, Nieves-Aldrey JL. Gall-Inducing insects and their associated parasitoid assemblages in the Forests of Panama. Neotropical Insect Galls: Springer; 2014. p. 465–96.

34. Holl KD, Lulow ME. Effects of species, habitat, and distance from edge on post‐dispersal seed predation in a tropical rainforest. Biotropica. 1997;29(4):459–68.

35. Wolstenholme B, Whiley A. Ecophysiology of the avocado (*Persea americana* Mill.) tree as a basis for pre-harvest management. Revista Chapingo Serie Horticultura. 1999;5:77–88.

36. Janzen DH, Martin PS. Neotropical anachronisms: The fruits the gomphotheres ate. Science. 1982;215(4528):19–27.

37. Hughes MA, Smith J, Ploetz R, Kendra P, Mayfield A, Hanula J, et al. Recovery plan for laurel wilt on redbay and other forest species caused by *Raffaelea lauricola* and disseminated by *Xyleborus glabratus*. Plant Health Progress. 2015;16(4):173–210.

38. Dyer LA. Tasty generalists and nasty specialists? Antipredator mechanisms in tropical lepidopteran larvae. Ecology. 1995;76(5):1483–96.

39. Niogret J, Epsky ND, Schnell RJ, Boza EJ, Kendra PE, Heath RR. Terpenoid variations within and among half-sibling avocado trees, *Persea americana* Mill. (Lauraceae). PLOS ONE. 2013;8(9):e73601.

40. Ingersoll CM, Niesenbaum RA, Weigle CE, Lehman JH. Total phenolics and individual phenolic acids vary with light environment in *Lindera* *benzoin*. Botany. 2010;88(11):1007–10.

41. Goralka RJ, Langenheim JH. Implications of foliar monoterpenoid variation among ontogenetic stages of the California bay tree (*Umbellularia californica*) for deer herbivory. Biochemical Systematics and Ecology. 1996;24(1):13–23.

42. González-Coloma A, Escoubas P, Reina M, Mizutani J. Antifeedant and insecticidal activity of endemic Canarian Lauraceae. Applied Entomology and Zoology. 1994;29(2):292–6.

43. González-Coloma A, Escoubas P, Lljide L, Mizutan J. Insecticidal activity screening of Japanese Lauraceae. Applied Entomology and Zoology. 1994;29(2):289–92.

44. Kishimoto-Yamada K, Kamiya K, Meleng P, Diway B, Kaliang H, Chong L, et al. Wide host ranges of herbivorous beetles? Insights from DNA bar coding. PLOS ONE. 2013;8(9):e74426.

45. Grubb P. Sclerophylls, pachyphylls, and pycnophylls: The nature and significance of hard leaf surface. Insects and the Plant Surface. 1986:137–50.

46. Vaz PP, de Souza PR, Alves FM, Arruda RdCdO. Cork-warts on leaves of Lauraceae: Confirming a suspicion. Plant Systematics and Evolution. 2018:1–7.
